# A data-driven perspective on the colours of metal–organic frameworks†

**DOI:** 10.1039/d0sc05337f

**Published:** 2020-12-28

**Authors:** Kevin Maik Jablonka, Seyed Mohamad Moosavi, Mehrdad Asgari, Christopher Ireland, Luc Patiny, Berend Smit

**Affiliations:** Laboratory of Molecular Simulation, Institut des Sciences et Ingénierie Chimiques, École Polytechnique Fédérale de Lausanne (EPFL) Rue de l'Industrie 17 CH-1951 Sion Switzerland berend.smit@epfl.ch; Institute of Mechanical Engineering (IGM), School of Engineering (STI), École Polytechnique Fédérale de Lausanne (EPFL) CH-1015 Lausanne Switzerland; Institut des Sciences et Ingénierie Chimiques, École Polytechnique Fédérale de Lausanne (EPFL) Rue de l'Industrie 17 CH-1951 Sion Valais Switzerland; Institute of Chemical Sciences and Engineering, École Polytechnique Fédérale de Lausanne (EPFL) CH-1015 Lausanne Switzerland

## Abstract

Colour is at the core of chemistry and has been fascinating humans since ancient times. It is also a key descriptor of optoelectronic properties of materials and is often used to assess the success of a synthesis. However, predicting the colour of a material based on its structure is challenging. In this work, we leverage subjective and categorical human assignments of colours to build a model that can predict the colour of compounds on a continuous scale. In the process of developing the model, we also uncover inadequacies in current reporting mechanisms. For example, we show that the majority of colour assignments are subject to perceptive spread that would not comply with common printing standards. To remedy this, we suggest and implement an alternative way of reporting colour—and chemical data in general. All data is captured in an objective, and standardised, form in an electronic lab notebook and subsequently automatically exported to a repository in open formats, from where it can be interactively explored by other researchers. We envision this to be key for a data-driven approach to chemical research.

## Abstract 2

Colour is at the core of chemistry and has been fascinating humans since ancient times.

## Graphical abstract

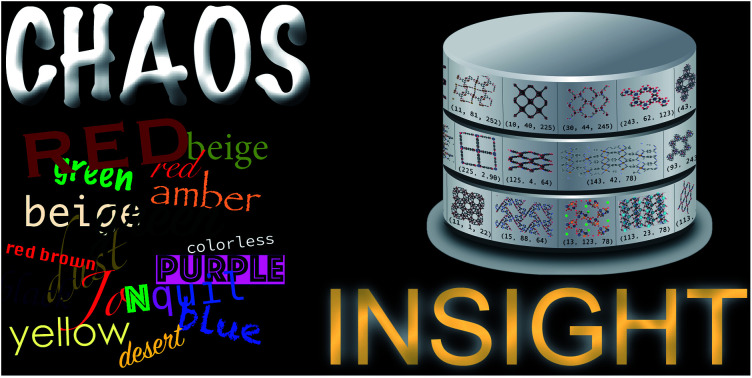

## Introduction

Colours have been attracting the attention of humans for a long time and are one key aspect that makes chemistry interesting.^1^ Chemists have some intuition within compound classes how they can tune the colours. For organic compounds, group-contribution methods like the Woodward rules found wide acceptance.^2^ In other cases, chemists might have an intuition if certain transitions are allowed or forbidden, *e.g.*, based on Laporte's rules in metal complexes.^3^ However, colours can be influenced by very subtle effects which led some authors to conclude that “*the prediction of the colouring properties of yet unsynthesised compounds is a very risky business which still remains in the realm of art rather than of science*”.^1^ Even though we can use quantum chemical calculations to estimate band gaps,^4,5^ or even the full dielectric function and absorption spectrum,^6,7^ those calculations are computationally prohibitive for large unit cells and require careful consideration of non-ideal effects such as defects.^8^

In this work, we focus on the colours of metal–organic frameworks (MOFs). MOFs are crystalline materials with a unique chemical tunability.^9^ By changing the metal node and the organic linker, we can synthesise millions of possible materials. These materials have many interesting applications, ranging from gas storage and separations,^10^ (photo)catalysis,^11^ to sensing,^12^ and luminescence.^13^ The chemical toolbox, like the substitution of metal and linkers, with which the optical properties of MOFs can be tuned has been exploited in several works in the past.^14–17^ For applications of MOFs that rely on the optical properties of MOFs (*e.g.*, photocatalysis, sensing, luminescence, optoelectronics) selecting a MOF with the right colour is important. Additionally, the colour is also of importance to assess the success of their synthesis, work-up, and activation—this is one of the reasons why the colour of the products is usually reported in method sections.

In the Cambridge Structure Database (CSD)^18^ some experimental groups report together with the structure also the colour of the MOF (circa 9000 structures of the more than 100 000 structures^19^ in the MOF subset^20^ of the CSD). In this work, we show that this data can be harvested using machine learning to arrive at a tool that can efficiently predict the colour of a MOF. It is important to realise that at present the CSD is the only data source that we have at disposal to develop a model to predict optical properties of MOFs as we are not aware of any large dataset that reports reliable optical gaps or other optical properties of MOFs. In this database, the amount of data is relatively small, and the naming of colours is unfortunately rather subjective. From a machine learning point of view, such little and subjective data pose an interesting scientific question, which we attempt to answer here: how we can use such data to build a surrogate model for the optical properties of MOFs, what we learn by doing so, and more importantly, how could we improve this situation moving forward?

The idea of machine learning is that similar MOFs will have similar colours. However, the concept of similarity depends on the property we are interested in.^21^ It is therefore important to develop a machine learning approach that closely follows the chemical intuition that chemists have developed over many decades in what makes two MOFs give a similar colour. Hence, in our machine learning approach, we ensure that our descriptors can capture all possible ways of electronic transitions that can lead to different colours. This includes information about the metal to be able to describe metal-centred transitions, information about the ligands, and the functional groups to be able to describe ligand-centred transitions, as well as information about the interaction between metal and ligands to be able to describe metal–ligand or ligand–metal transitions. Only if we ensure that our featurisation has the expressivity to describe these phenomena efficiently and effectively, we can combine our chemical insights into what is important in determining a colour with a relatively small database of materials of which we know the colour.

The other interesting point is that a colour can be a well-defined property. One can precisely specify the colour of a material by its tristimulus values in some colour space, similar to how the human eye perceives colour with three types of cones.^22^ Such high-quality data for MOFs is scarce. In fact, the data in the CSD are the (subjective) names given by the groups that have synthesised the material. If the name is blue or red, it might be clear to which colour (range) the group is referring to, but if the given colour of a MOF reads “straw yellow” or “claybank” it becomes more difficult as we have to take into account that every person has a distinct perception of colours. This is, the colour intended can not easily be inferred from the reported colour name.^23^ To address this issue we have conducted a survey that allows us to map the colour names to a distribution of coordinates in a colour space (representing the estimates of the likelihood of the colour intended) and also gives us an idea of the perceptive spread for different colour names. This survey also revealed that the current way colour is reported in chemistry is inadequate. It hampers data-driven approaches to chemistry and also limits the reproducibility.

In addition, we also present some very practical tools. One is a web application that allows uploading a structure and the app predicts the colour together with an uncertainty estimate. The other app allows measuring the colour of a MOF based on a picture of the synthesised material, such that chemists can report the tristimulus values (like coordinates in RGB space) together with their favourite name for the colour of the material. We hope that the latter can help to improve the reporting of colours in chemistry. Furthermore, we demonstrate how an electronic lab notebook can be used to capture and share data in standardised and digital from, enabling interactive, and digital, Supporting Information (SI) documents† (see https://go.epfl.ch/colourSI for an example) that are much more accessible to data mining efforts than classical SI documents in portable document format (PDF). Importantly, these interactive electronic SI documents are not limited to our particular application. We envision them to be an important part of chemical publishing in the future.

## Colours and their perception

**Fig. 1:**
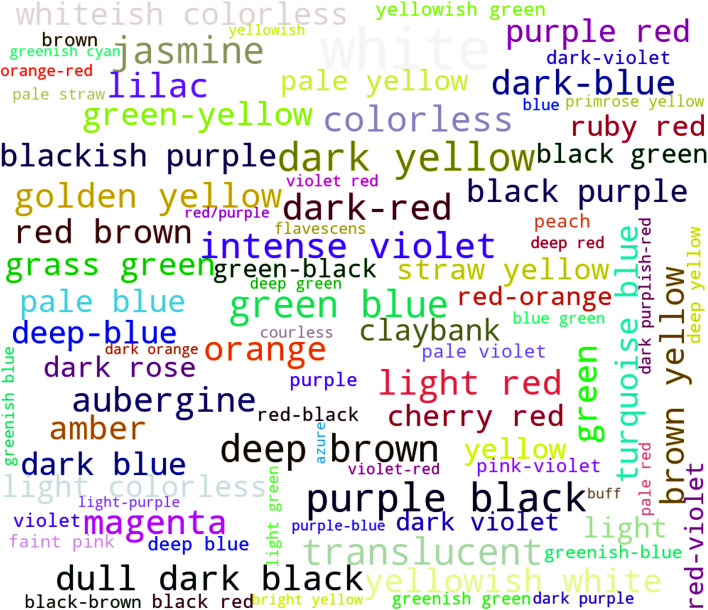
Words that are used in the CSD to describe colours. The words are coloured using the median colour (unweighted average in RGB space) from the survey. The size of the words is proportional to their frequency. Figure generated using the WordCloud library.^24^

Fig. 1 illustrates some words that are used in the CSD to describe the colour of MOFs. From a machine learning point of view, such discrete and subjective data are of limited use. First, using the names of the colours we cannot easily encode that confusing orange with yellow is not as bad as confusing black and white. Further, if for some colours the spread of the perception is wide, we also would not be surprised if the model is unsure about the colour.

The perception of colour has already been studied in a widely known survey conducted by Randall Munroe (creator of the webcomic xkcd).^25^ Previous approaches for mapping colours to colour names were mostly focused on human-curated dictionaries. In contrast to that, for Munroe's dataset, nearly half a million participants named colours which they were shown. The large number of participants made this dataset an important reference for data-driven approaches to natural language processing.^23,26–30^ For our purpose, we are interested in the reverse question, mimicking how chemists would try to assess if they successfully reproduced a colour reported in the literature: given a name what is the colour one would associate with this name and how large is the spread of these colours associated with a colour name? This question, that is also important for natural language understanding, is less widely studied than the reverse one,^23,31,32^ and information we cannot easily obtain from the xkcd survey. First, because one-third of the colours that are used in the CSD to describe the colour of a MOF are not represented in the xkcd survey and, second, because we are also interested in the spread of responses to get a baseline of how well we can expect our model to perform in different parts of colour space. To obtain some insight into this question we carried out a survey resulting in 4184 assignments of colours to one of the 162 names that occurred in the CSD for colours of MOFs. In the ESI,† the details of the survey are given. Note that in contrast to other works^23,30,33,34^ based on the xkcd survey we did not attempt to build a general model that maps colour names to the tristimulus coordinates of the intended colour but rather want to infer the likelihood of the intended colour for all colour names that are used for MOFs in the CSD.

## Perceptive spread of colours and the current way of reporting colours

**Fig. 2:**
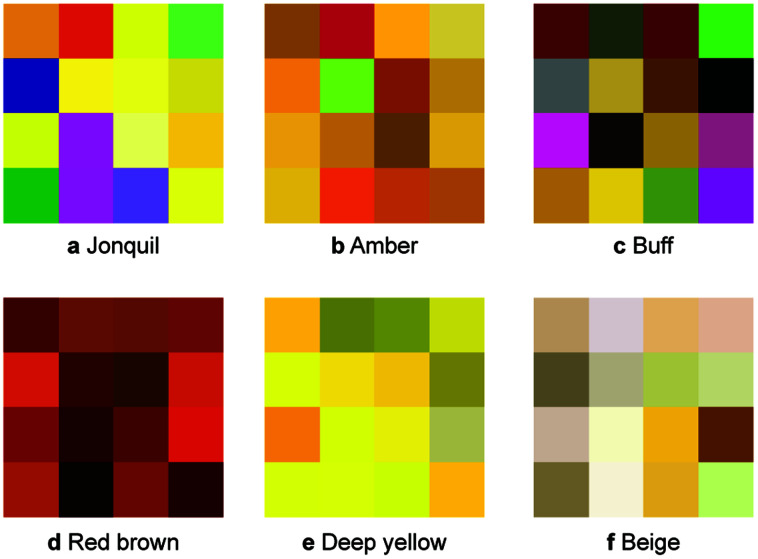
Examples of the spread of some colours in our survey. The plots show nine random samples from the survey results for each colour name. Note that for this figure we already applied a threshold on the minimum and maximum time for picking the colour. That is, for each of the colours the participants required more than five, but less than 80 seconds to select it. For some colours like “*jonquil*” and “*buff*” the spread is so large (presumably due to linguistic barriers) that we cannot use the data for meaningful training.

The first question to pose is if our survey results can give us meaningful insights, *i.e.*, whether the statistics are good enough, and our data are representative. One way to estimate this is to compare our results with the ones from the xkcd survey for the colours that overlap between both surveys. That is, we ask if the median of the colour distribution obtained from our survey corresponds to the colour that has been given this name in the xkcd survey. From ESI Fig. 8† we see that our findings, in general, agree well with the ones from the xkcd survey. Still, when we analyse the individual submissions, we find that there is a considerable spread in the colours the users selected—even after filtering out outliers (for example, we discarded submissions if the colour was picked in less than 5 s). In Fig. 2 we show the spread in the responses for some colours. It is instructive to quantify the spread in colours. A widely used metric to quantify differences between colours is the 
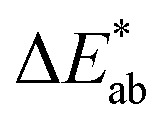
 score (using the CIEDE2000 formula),^35^ which takes into account that the human eye is more sensitive to certain colours. For professional prints one typically expects^36^
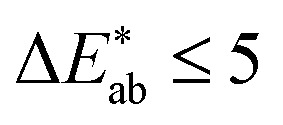
 and a 
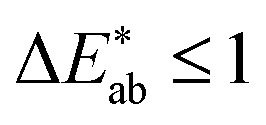
 is said to be undetectable for the human eye.^37^ Notably, we found in our survey only black to have a median 
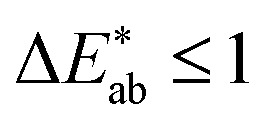
 and only five colours in total (black, red, white, whitish colourless, yellow, corresponding to less than four per cent of all colours in our survey) have a median difference between the responses in the survey that would satisfy common printing standards. The overall median of the differences is approximately 10 (mean: 12). This implies that if we filtered out all high variance colours, we would have too little data to train our model (see ESI Fig. 10†). Still, we observe that for some colour names like “jonquil” or “buff” the spread is so large that it is not practical for use in training a model (more discussion in Section 2 of the ESI†).

For our current study, our simple survey allows us to replace the discrete names, like “cherry red”, with a distribution of colour coordinates in some colour space which we can use as data in our machine learning approach. This approach is sufficient to demonstrate the potential of machine learning in predicting colours. But the fact that we have to use a survey to quantify colour does illustrate that the current way of reporting colours is inadequate—especially in the face of the variance which we observe in the survey results. Clearly, the problems with colour reporting go beyond natural language. For example, the concept of colour constancy (the way in which our brain resolves inconsistent colour signals when the illumination changes) was suggested as an explanation for the different colours humans perceived for “the dress” that went viral in 2015.^38,39^ Since in science we want to record information in a way that is invariant to subjective perception, we need a new way to record and report colours in chemistry.

## Colour reporting and integration with an ELN

**Fig. 3:**
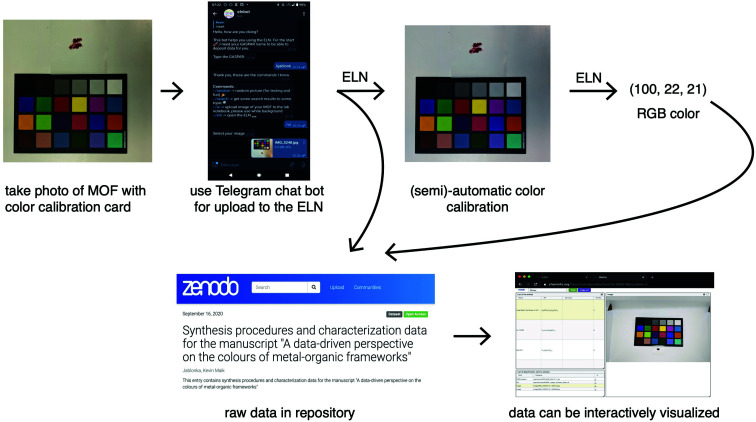
Schematic illustration of the semi-automatic colour calibration and subsequent publication of the raw data using the ELN. The user can take a photo with his or her phone and use a chatbot, or simply drag and drop, to deposit it in the ELN. This permanently preserves rich, and digital, information about the colour and morphology of the material that we also deem to be useful when the synthesis is replicated at some later point in time. This is facilitated by the fact that samples from the ELN can be exported with all characterisation data to Zenodo. A separate website, that reuses the data visualisation modules from the ELN, can be used to visualise the data deposited on Zenodo (here https://go.epfl.ch/zenodo_colorpaper).

For testing of our machine learning approach with some recently synthesised MOFs, we used a more objective and accurate way of recording colours. The idea is to take a photo of the material together with a colour rendition card. Such an image can then be automatically uploaded to an electronic lab notebook^40^ (ELN, see Fig. 3). This image can then, with all the characterisation data, be shared in digital, and standardised, form *via* a repository from where it can be accessed for data mining. A dedicated website can be used to visualise the data deposited in the repository (using the same code that is also used for data visualisation in the ELN). Importantly, our ELN makes it possible to perform this export and publication of findable, accessible, interoperable and reusable (FAIR)^41^ data for any kind of characterisation method and not only for this specific case. For example, the repository entry for this work also contains the X-ray diffraction patterns, thermogravimetric analysis or UV/Vis spectra for some materials.

Since the images we take of the MOFs also contain a colour rendition chart, we can perform colour calibration. By means of the colour calibration, the colour profile can be standardised, which can then be harnessed for more accurate colour measurements. In principle, one could also use a spectrophotometer to quantify the colour of a material. We decided to use images as we found it to be faster (also for small amounts of activated compounds). Moreover, the image records additional important information like the morphology, or reflexive properties, of the sample. Ideally though, one would record as much information as possible.

To facilitate this first step towards a good practice of accurate reporting of the colour of a material we have developed a web application. Our web application (https://go.epfl.ch/colorcalibrator) uses a fully automatic routine that automatically detects the colour rendition chart.^42^ The user only needs to upload a photo of the MOF with a colour rendition chart and select an area over which the colour averaging should be performed.

The implication of this infrastructure, which directly connects the capture of the data with the publication, is that if it were used by many groups, we would create much more valuable data that would make works more comparable and machine learning methods thrive. Also, we could replace Supporting Information documents in portable document format (PDF) with data that is alive and reusable. As we did for this article, researchers could just report the digital object identifier (DOI) for their repository entry instead of, or in addition to, providing the PDF.

## Model development

To build a robust model it is instrumental that two materials that have structures that are close in terms of their colours are also close in terms of the descriptors. The intuition here is to encode the nodes, the linkers, and the functional groups separately by using correlations on a structure graph coloured with some chemically sensible heuristics such as the electronegativity or polarisability. This is, the model will be able to recognise “colouring” functional groups by their characteristic autocorrelation functions. To achieve this, we use the revised autocorrelation (RAC) function^43^ formalism which was used in the past to predict electronic properties of metal complexes^44,45^ and recently adapted for MOFs.^46^ RACs are discrete correlations between heuristics (*e.g.*, Pauling electronegativity) of atoms on the structure graph which are then pooled together for small fragments of different size. For MOFs, we calculate those descriptors separately for linkers, functional groups and the nodes. We augment this set of features with additional descriptors for the linkers, such as the number of aromatic rings, aromatic bond, or double bonds, that we anticipate having a high association with the colour of the compound (see Section 4.1 of the ESI† for more details).

For making the predictions based on those descriptors, we use a gradient boosted decision tree (GBDT) model, which is an ensemble of decision trees that are iteratively fitted on the residual of the previous decision tree to predict tristimulus values that are close to the median colour coordinates we extracted from our survey for the colour name of a given MOF. We found this method to perform best across a range of other models we tested (see ESI Section 4†). We built our model based on 6423 structures from the structures in the MOF subset with a colour attribute, from which we dropped duplicates to avoid data leakage (see ESI Section 3†).

## Model evaluation

**Fig. 4:**
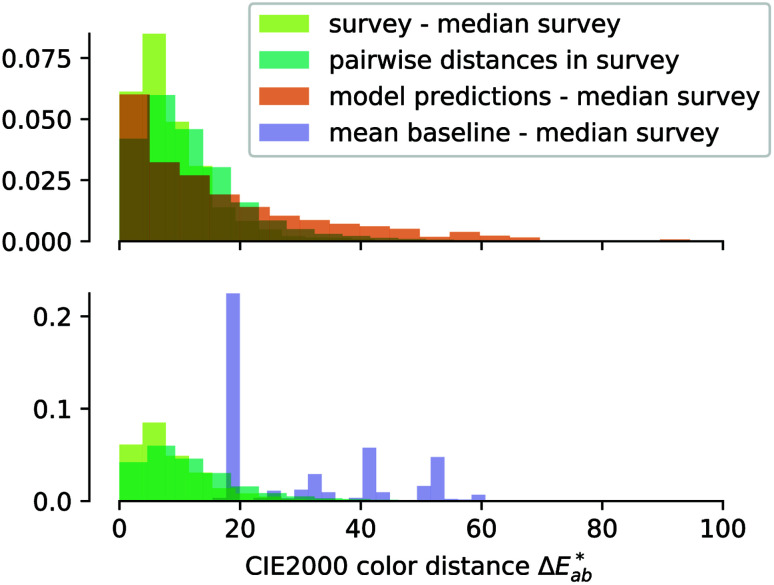
Error distribution for our survey (pairwise differences and mean difference to the median, in light and dark green), our model (orange), and a baseline model (purple). For the survey, we weighted the colours by their frequency in the MOF dataset. The horizontal axis shows the colour distance 
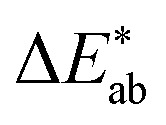
 and the vertical axis shows the density.

To allow for evaluation of our model, we held out a test set of structures which we did not use for training or hyperparameter tuning. Our model achieves a good predictive performance for those structures, as shown in some examples of randomly picked predictions in ESI Fig. 13† and in numerical metrics [mean absolute error (MAE) = 0.14 (0.13, 0.15), *r*^2^ = 0.54 (0.50, 0.57), a mean baseline gives MAE = 0.31 (0.31, 0.32), *r*^2^ = 0 (0, 0)] calculated over the full test set. One may wonder how these numbers, *i.e.* the performance of our model, compares to the perceptive spread we observed in the survey. Above, we calculated the 
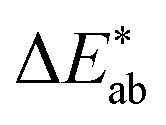
 differences for each colour in our survey and did the same for our (baseline) models (Fig. 4). That is, a small 
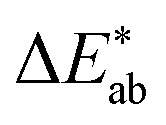
 indicates that the colour predicted by our model is close to the median tristimulus values that we extracted for a given MOF colour name using the survey. We observe that the distribution of colour differences for our model has a more pronounced tail of larger differences, and also a larger median of 17 (16, 18), compared to a median of 10 (mean: 12) for the in-survey differences. But the fact that our median is close the median of the in survey errors reflects that our model is mostly limited by the inherent variance of the data (given that the learning curves in ESI Fig. 20† did not saturate). Interestingly, about 28% of our predictions are less than 5 
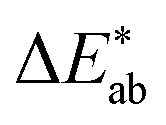
 units (the tolerance used for printing) from the median of the survey.

By training models to predict also the quantiles, *i.e.*, the error bars around the median, we observed that the model often is uncertain about the intensity of the colour, *e.g.*, the 90th percentile is frequently close to colourless. This points to another problem with the reporting of colours—the colour string often gives no information about the chromatic intensity. Indeed, if we analyse the colour names in the CSD we observe that only one-third of all colour strings have intensity information such as “light” or “dark” in the name—and even then the exact position on the continuum of intensities is not well-defined.

## Test on experimental compounds

For some compounds that our experimental colleagues had recently prepared for testing of our model, we recorded the colour as outlined in Fig. 3 and used our model to predict the colour. For all compounds, we ensured that we include no other too similar compound within some distance in the feature space in the training set (see ESI Section 6.2†).

**Fig. 5:**
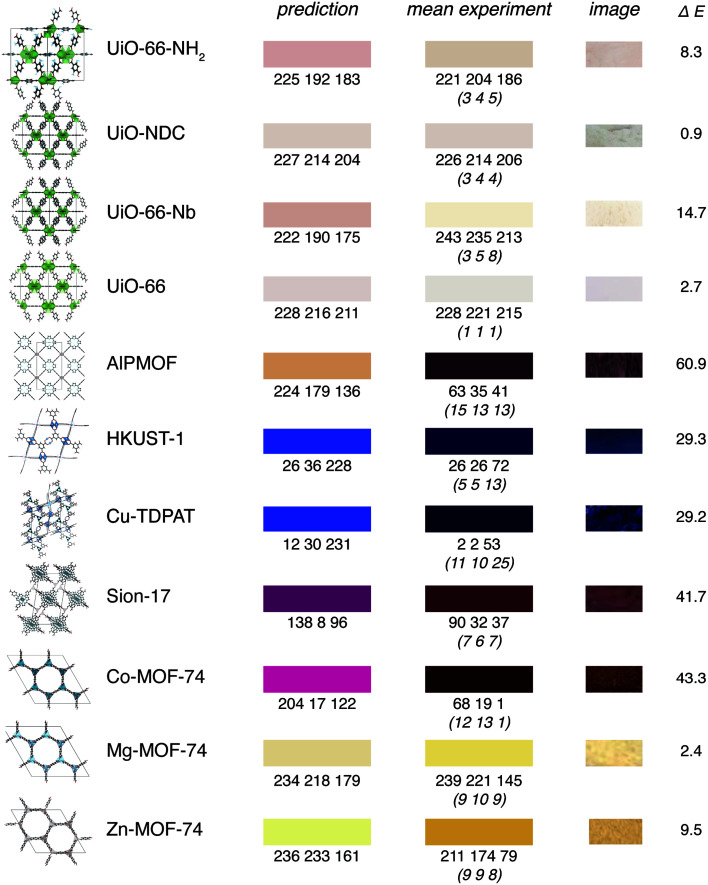
Examples for predictions on experimental structures that are not part of our training set. The left patch shows the predictions of our model, the middle patch shows the average colour of the image, right patch shows a colour-calibrated image of the compound (using the Vandermonde method, oblique numbers in parentheses indicate the standard deviation). We excluded all structures from the training set that are within 0.02 Manhattan (*l*_1_) norm from the descriptor of the experimental structure (note that the exact geometry does not play a role in our featurisation, only the bonding graph is used to compute the features).

We can observe that even though the predictions might be quantitatively not perfect, given the uncertainties in the way colours are reported in the CSD, our results are certainly encouraging (Fig. 5). This is also reflected in the fact that the mean absolute error of our model is close to the mean variation of the colours in our survey.

In particular, we capture many interesting trends. For example, our model recognises that the addition of an amino group leads to a redshift for UiO-66. Likewise, we can analyse the influence of metal substitutions, *e.g.*, the doping of UiO-66-NH_2_ with Nb leading to a redshift as described by Syzgantseva *et al.*^47^

## What did the model learn?

Machine learning is often seen as a black box, in which we replace our chemical knowledge and intuition by plain statistics.^48^ However, we can analyse the importance of the different features, and this feature analysis will tell us what the most important features are. Here, we are interested in which features make a MOF red (R), green (G), or blue (B) for our model.

**Fig. 6:**
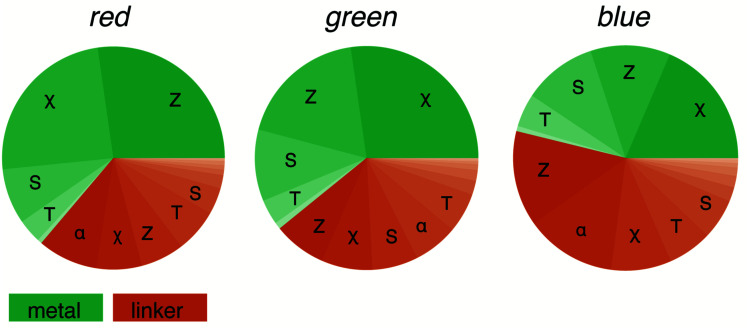
Feature importance as a function of the RGB colour channel. The colour of the pie slices separates metal from linker contributions. The linker contributions are more important for the blue channel (red absorption). RACs features are grouped according to the heuristic used in their construction: *χ* electronegativity, *T* topology (number of bonds), *S* covalent radius, *α* polarisability, *Z* atomic number.

For this, we split the features into metal-centred and linker-centred contributions and evaluate their absolute importance as a function of the colour channel. Fig. 6 shows that for our model the characteristics of the metal is most important for the red colour channel. For the blue colour channel, being more relevant for absorption at longer wavelengths (the complementary colours are absorbed), the linker chemistry is more important for our model. But in no instance, the model relies solely on metal or linker and descriptors (for discussion of the interactions between the features see the ESI Section 7†). This supports the notion that for visible-light-driven applications of MOFs the interaction between metal and linker is important (linker-to-metal-cluster charge-transfer mechanism, LCCT).^49^

**Fig. 7:**
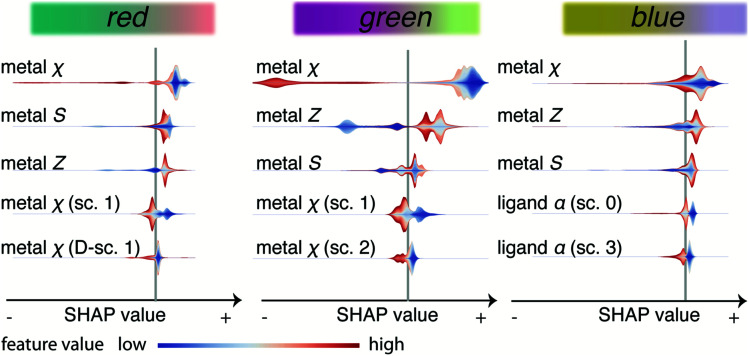
Shapley additive explanations for the five most important features for every colour channel. The abscissa shows the SHAP value, *i.e.*, the impact on the output for the model. For example, a high value for red means that the model will output a higher value for the red RGB channel. The vertical grey line shows the baseline prediction, *i.e.*, with uninformative features. The feature values, like the electronegativity of the metal, are shown with colour-coded violin plots. The colour-coding gives the value of the feature (with respect to the distribution of all features in the dataset) and the width of the violin indicates the distribution. Abbreviations for RACs heuristics: *χ* electronegativity, *T* topology (number of bonds), *S* covalent radius, *α* polarisability, *Z* atomic number. The scope of the RACs, *i.e.*, the coordination shells which are considered for the correlation, is abbreviated with sc, *D* indicates difference RACs. The colourbars for each colour channel illustrate how they affect the colour. For this, we fix the values of the other two colour channels fixed at the mean value from the training set and linearly vary the colour of one RGB channel from the minimum (left) to the maximum value (right).

Our models indicate that linker modification is important to tune absorption in the visible regime, which triggers an important practical question. Can our model give us some insights about how we can tune the material to steer the optical response? We can get more insight into the direction in which the features influence the colours by analysing Fig. 7. This figure lists the five most important features (biggest slices in Fig. 6). This graph gives the SHAP value of each property, which is a measure for the impact on the output of the model, on the *x*-axis. A high absolute SHAP value means the feature has a large impact, which can be positive (increasing the R, G, or B values) or negative. For each material and feature, we get a SHAP value, and the colour coding indicates whether the feature value is high or low. For example, the violin plot for the red colour channel shows that metals with a low *χ* (blue), all have a positive SHAP value, indicating a low electronegativity leads to a higher output on the red colour channel.

Overall, we observe that the colourfulness primarily depends on the position of the metal (metal *Z*, *χ*, *S*) in the periodic table—broadly speaking, a high electronegativity tends to decrease the output on all colour channels (especially red and green). Similarly, we see increasing atomic number leading to increased output on all colour channels—but all those trends are not simple monotonic relationships. These observations can be thought of as a refined version of previous suggestions that an electron-rich metal centre (soft, *i.e.*, low *χ* and large *S*) can be used to decrease the bandgap.^50,51^ But for our model, this does not happen equally for all colour channels. For example, for the blue colour channel we see strong interaction effects of the metal features with linker features. For instance, high and low values of *χ* and *Z* give rise to the same SHAP value and the impact on the output will depend on the value of linker features for a given value of the metal features. We suppose that this reflects that for an LCCT transition the energy levels of the metal cluster and the linker need to be properly aligned.

One interesting case to understand how the model learns is the case of HKUST-1 for which Müller *et al.* have shown that the green-blue colour that is typically observed for powders of this material is due to d–d transitions in defective paddlewheels (in perfect structures, the selection rules for the 
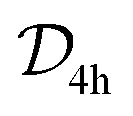
 symmetry lead to only weak transitions).^8^ Not surprisingly, our model predicts a blue colour for this MOF as this is the colour reported in the CSD for HKUST-1 (CSD reference codes BODPAN, FIQCEN, but we excluded it from the training set). This is likely also one of the reasons why we predict blue for UMCM-152 (CSD reference code ANUGIA) which is reported as blue (dark-purple after drying) in the paper^52^ but as colourless in the CSD. Predicting colours that are due to defects would not be possible in a first-principles approach based on idealised crystal structures (for which transitions might be forbidden due to selection rules), but is, as we show, possible in a data-driven approach. Since the model learns chemical similarities in some descriptor space it will predict similar colours for similar MOFs that might have similar defects—which might not be directly clear from the crystal structure, as in the case of HKUST-1.

## Conclusions

Predicting the colour of synthesised compounds was long deemed to be a “risky business”. In this work, we showed that it is possible to leverage a relatively small dataset of subjective and categorical assignments of colours to MOF structures to build a predictive model that outputs colours on a continuous scale. Furthermore, we show that the reasoning of our model is chemically meaningful, for example, recovering many aspects of an LCCT transition and recovering trends like colour changes for substitution of metal or ligand.

In the process of building our model, we uncovered inadequacies in the way colours are reported. The common practice is to simply provide a name of the colour. Our simple survey shows, for example, that if one reports that the colour of a compound is beige, there is a large variation in colour different people associate with the name beige. In fact, that the variance of perception for most colours is above common tolerances for colour reproduction—ultimately limiting the learning our model. To remedy this, we propose a simple way to improve the reporting of colours. One can only imagine how much we could improve this model if we would have a large dataset of such high-quality data at disposal. Future work needs to focus on creating large scale, objective datasets mapping chemical structures to their colours.

Importantly, colours are only one example where chemical reporting can be improved. Generally, we envision that all reporting should happen in a digital, standardised and unique way. In this work, we provide an example of how this can be done using our electronic lab notebook (ELN). The idea is to take a picture with a smartphone of the sample together with a colour calibration card. This picture gets automatically uploaded into the ELN, and we have provided a tool that automatically recognises the calibration card and by clicking on the sample one can obtain the RGB value of the sample. In addition, from the ELN all data can be exported to a repository in a FAIR format, providing an alternative to the conventional SI in PDF.

Some of our ongoing work focuses on extending the set of characterisation techniques that are supported by our ELN to make this toolbox accessible to a wider group of chemists and materials scientists. Applying machine learning techniques to such standardised datasets might help us then to extract hidden, tactic, knowledge from this data.

## Method

### Online survey

We developed a custom tool (http://go.epfl.ch/colorjeopardy), based on the Plotly Dash^53^ Python framework, to conduct the online survey. Users were presented a random colour string (that was used to describe the colour of a MOF in the CSD) and then could use a colour picker to select the colour that most represents this colour string for them. We recorded the colour picked with the sRGB coordinates and the time the users took to select the colours. Note that our setup, similar to the one of the xkcd survey, did not ensure that the users see controlled colours (*e.g.*, on a colour-calibrated monitor). The code is available under MIT License on GitHub (https://github.com/kjappelbaum/colorjeopardy, DOI: 10.5281/zenodo.3831841). The survey results are deposited on Zenodo (https://zenodo.org/record/3831845). Note that since the survey did not collect any personal information, no approval from the institutional review board was required.

### Colour calibration

More details can be found in Section 8 of the ESI.† The app is deployed at http://go.epfl.ch/colorcalibrator (code is available on GitHub at https://github.com/kjappelbaum/colorcalibrator).

### Featurisation

To numerically encode the MOF structures, we used RACs, as recently implemented for MOFs in the molSimplify code.^46,54^ Additionally, we used the SMILES strings of the linkers, as determined using the MOFid package,^55^ to calculate features that describe the chemistry of the linkers, focusing on aspects that we deem to be important for the colour of compounds—such as the size of the aromatic system, the number of double bonds or functional groups such as amides or carbonyls using Open Babel.^56^ We *z*-score standardised the features based on the mean and standard deviation of the training set. All preprocessing was performed using the scikit-learn Python library.^57^ The feature arrays are deposited on Materials Cloud archive (https://archive.materialscloud.org/record/2020.163).

### Machine learning

We used the LightGBM implementation of GBDTs, which implements techniques that greatly expedite the training for high feature dimensions and large datasets.^58^ To obtain prediction intervals, models were trained using the quantile loss function (0.5, *i.e.*, the median prediction corresponding to the mean absolute error loss). For hyperparameter optimisation, we used a Bayesian approach with Gaussian processes as surrogate models (details like the parameter ranges are provided in the ESI Section 4†). For efficiency reasons, we used the same hyperparameters for every colour channel. To calculate the CIE2000 colour differences we used the implementation in the colormath Python package.^59^ Machine learning experiments were tracked using comet.ml (https://www.comet.ml/kjappelbaum/color-ml?shareable=jfE6okDmxlnYimYFFnsJcMCO6) and wandb (https://app.wandb.ai/kjappelbaum/colorml). The codes for the models (also for the failed attempts) and the analysis is available on GitHub (https://github.com/kjappelbaum/colorml). The numerical metrics that we report are calculated with respect to the median colour labels that we found by mapping the colour strings in the CSD to a distribution of colours through our survey. Confidence intervals (reported in parenthesis following the mean) are determined using the bootstrapping technique, typically with 5000 samples. To stabilise the model (reduce the variance), we employed bagging, *i.e.*, the model was trained on 30 different bootstraps of the training set and the final prediction is the mean prediction of the sub-models.

For the validation of our model, we dropped duplicates, structures that are similar to our case studies, and split the database in a training set (90%) and a test set (10%) using iterative stratification.^60^ For doing so, we binned each colour channel into three equally sized bins and then applied the iterative stratification algorithm to ensure that the train and test sets contain the same proportions of regions of the colour space.

The model is deployed as a web app with the name “MOFcolorizer” at https://go.epfl.ch/mofcolorizer (the code for this app is available on GitHub, https://github.com/kjappelbaum/mofcolorizer). In addition to the explicitly mentioned codes, our work made use of the following Python packages: colour-checker-detection,^61^ colour,^62^ crystal_toolkit,^63^ dokku,^64^ flask,^65^ gunicorn,^66^ iraspa,^67^ jupyter,^68^ matplotlib,^69^ numpy,^70^ OpenCV,^71^ pandas,^72^ Pillow,^73^ pymatgen,^74^ PyTelegramBotAPI,^75^ rdkit,^76^ scipy.^77^

### Feature importance analysis

For feature importance analysis, we used the tree SHAP technique, marginalising over the training set.^78^ We averaged over the feature importance for each estimator of the bagged estimator.

### Export of characterisation data

The data is captured *via* an ELN,^40^ for which parsers are being developed for the relevant experimental data (all code is part of the cheminfo GitHub organisation, http://github.com/cheminfo, for this work, we, for example, used the parser for powder X-ray diffraction data^79^). The parsed data (which is stored in a CouchDB database) is then exported using the RESTful Application Programming Interface (REST-API) restoncouch,^80^ with other sample information to Zenodo. Spectra are typically stored in JCAMP-DX format,^81,82^ molecules in mol format, and sample information with metadata in JavaScript Object Notation (JSON). The characterisation data is available on Zenodo (DOI: 10.5281/zenodo.4044212) and can be visualised using a view developed with the Visualizer library (https://go.epfl.ch/zenodo_colorpaper).^83,84^ Large parts of the code for this view are also used in the ELN itself (eln.epfl.ch).

## Conflicts of interest

There are no conflicts to declare.

## Supplementary Material

SC-012-D0SC05337F-s001
