# Supplementary material for: A data-driven perspective on the colours of metal–organic frameworks
Source: Chem Sci. 2020 Dec 28;12(10):3587–98. doi: 10.1039/d0sc05337f (PMC8179528; doi:10.1039/d0sc05337f)
Supplement: SC-012-D0SC05337F-s001 [file SC-012-D0SC05337F-s001.pdf]

Electronic Supplementary Material (ESI) for Chemical Science.
This journal is © The Royal Society of Chemistry 2020

Supplementary Information for: A data-driven perspective on the colours of metal-organic frameworks

Kevin Maik Jablonka[1], Seyed Mohamad Moosavi[1], Mehrdad Asgari[2,3], Christopher Ireland[1], Luc Patiny[4], Berend Smit[1]✉

[1] Laboratory of Molecular Simulation (LSMO), Institut des Sciences et Ingenierie Chimiques (ISIC), École Polytechnique Fédérale de Lausanne (EPFL), Sion, Switzerland
[2] Institute of Mechanical Engineering (IGM), School of Engineering (STI), École Polytechnique Fédérale de Lausanne (EPFL), CH-1015 Lausanne, Switzerland
[3] Institut des Sciences et Ingénierie Chimiques, École Polytechnique Fédérale de Lausanne (EPFL), Rue de l'Industrie 17, CH-1951 Sion, Switzerland
[4] Institute of Chemical Sciences and Engineering, École Polytechnique Fédérale de Lausanne (EPFL), CH-1015 Lausanne, Switzerland
✉ berend.smit@epfl.ch

Contents

1 Exploratory data analysis of the online survey **3**

2 Perceptive spread in colours **13**

3 MOF structures and colour labels **14**
3.1 Elemental composition of the dataset 14

4 Model architecture **15**
4.1 Features 15
4.1.1 Revised autocorrelation functions (RACs) 15
4.1.2 Additional linker features 16
4.2 Hyperparameter optimization 16
4.3 Colourspaces 17

5 Sensitivity to the cutoff in the variance in perceptive spread and using all datapoints from the survey as noisy labels **19**

6 Performance measurement **22**
6.1 Representative predictions 22
6.2 Test in experimental compounds 24
6.2.1 UiO−66−NH_2 24

6.2.2 UiO-NDC . . . 24
6.2.3 Mg-MOF-74 . . . 24
6.2.4 Co-MOF-74 . . . 25
6.2.5 Zn-MOF-74 . . . 25
6.2.6 Sion-17 (first reported in this work) . . . 25
6.2.7 HKUST-1 . . . 26
6.2.8 Cu-TDPAT . . . 27
6.2.9 AlPMOF . . . 27
6.3 Examples of errors . . . 28
6.4 Baseline models . . . 30
6.5 Permutation test . . . 33
6.6 Learning curve . . . 34

7 Feature importance analysis **35**
7.1 SHAP interaction values . . . 35

8 Colour calibration **39**
8.1 Semi-automatic colour calibration . . . 39
8.1.1 Caveats . . . 39
8.2 Implementation . . . 42
8.2.1 Python implementation . . . 42
8.2.2 JavaScript Implementation . . . 43

9 Telegram chatbot **44**

10 Web app **47**

1 Exploratory data analysis of the online survey

We invited our followers on Twitter to participate in the survey, and also shared the link to the survey with all members of the schools of basic science and engineering at EPFL. In total, 4184 colours were picked by the participants. Participants could take the survey as often as they wished.

In Supplementary Figure 1 we show the distribution of the time the user took to pick a colour in our survey. The distribution is skewed, with a mean of 34.8 s and a median of 21.6 s. The maximum is 4113 s, the minimum is 0.76 s.

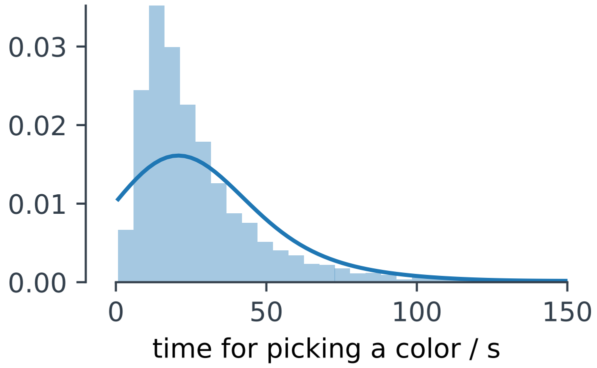

Supplementary Figure 1 | Distribution of the time the users needed to pick a colour. The smooth line represents a Gaussian kernel density estimate.

From the analysis, we eliminated 309 entries for which the users took less than 5 s (ca. 2.6 %) or more than 80 s (ca. 4.9 %).

It is interesting to analyse for which colour strings the users took most or least time. These are shown in Figure 2, where it is observable that there is a striking spread in the median time participants took to select the colour depending on the colour name.

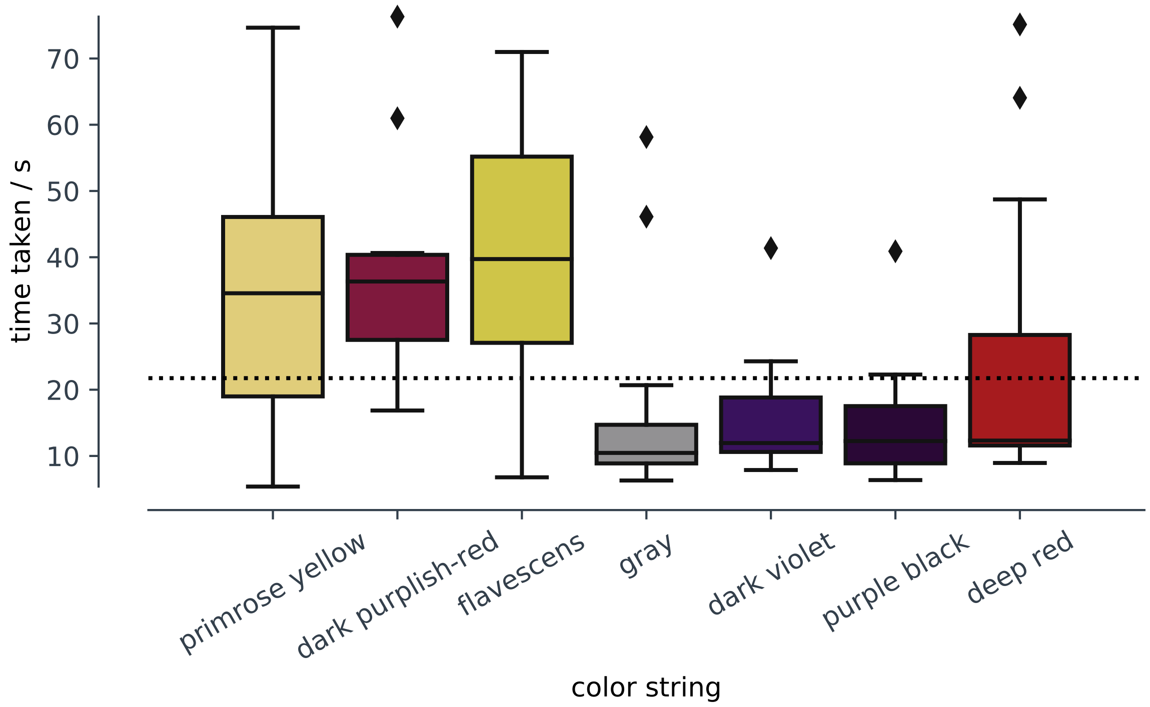

Supplementary Figure 2 | Boxplots for the time taken to select the colours. The dotted line shows the median. Bars coloured using the median colour (unweighted median in red, green, blue (RGB) space).

For use in scholarly communication, the name must be unambiguous. As a surrogate for the ambiguity we use the variance of the colour picks in RGB space. Care has to be taken here as the sensitivity of the human eye is not uniform across the whole colour space, wherefore we also calculated standard deviations that attempt to take this into account by weighting the channels with the luma weights, i.e., the red channel with 0.299, the green channel with 0.587, and the blue channel with 0.114.
We furthermore removed outliers using a $z = 2.5$ threshold on each colour channel.

We show the colours with maximum and minimum standard deviations in RGB space in Supplementary Figure 3. Both in the unweighted and weighted measurement yellowish colours show a high standard deviation.

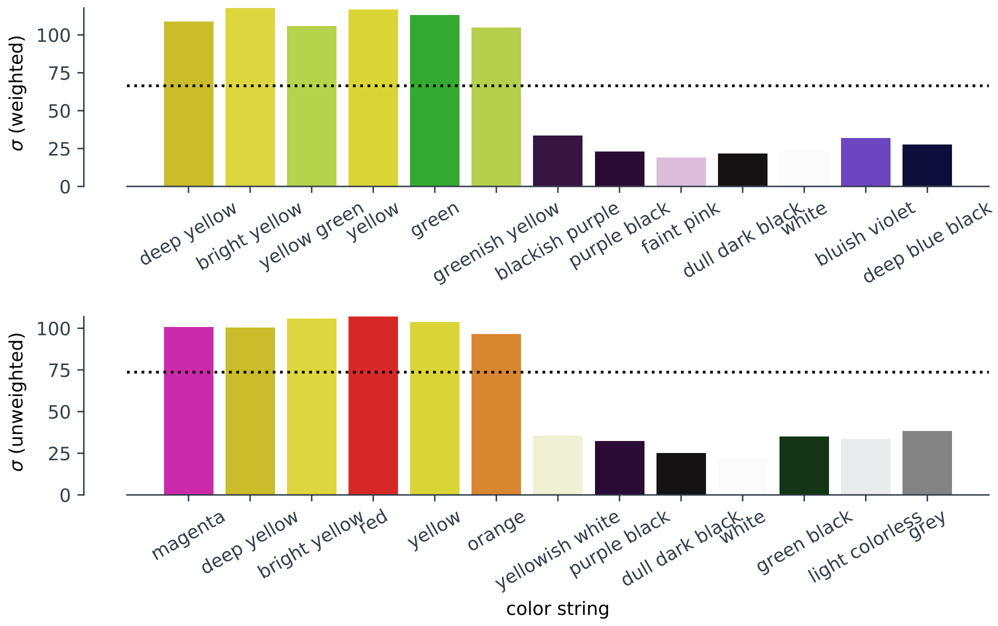

Supplementary Figure 3 | Maximum and minimum weighted and unweighted standard deviations in RGB space. Bars coloured in the median colour, the dotted line indicates the median.

One can get a good intuition for the choices of the participants by showing the choices for each colour term next to each other, as shown in Supplementary Figures 4–6. Interestingly, it is observable that some colour names like amber, that are less widely used, show a high variance. This is likely due to a linguistic problem. But also for more common colour names such as "yellow red" or "deep yellow" we observe a considerable variation in the colours that were picked by the users.

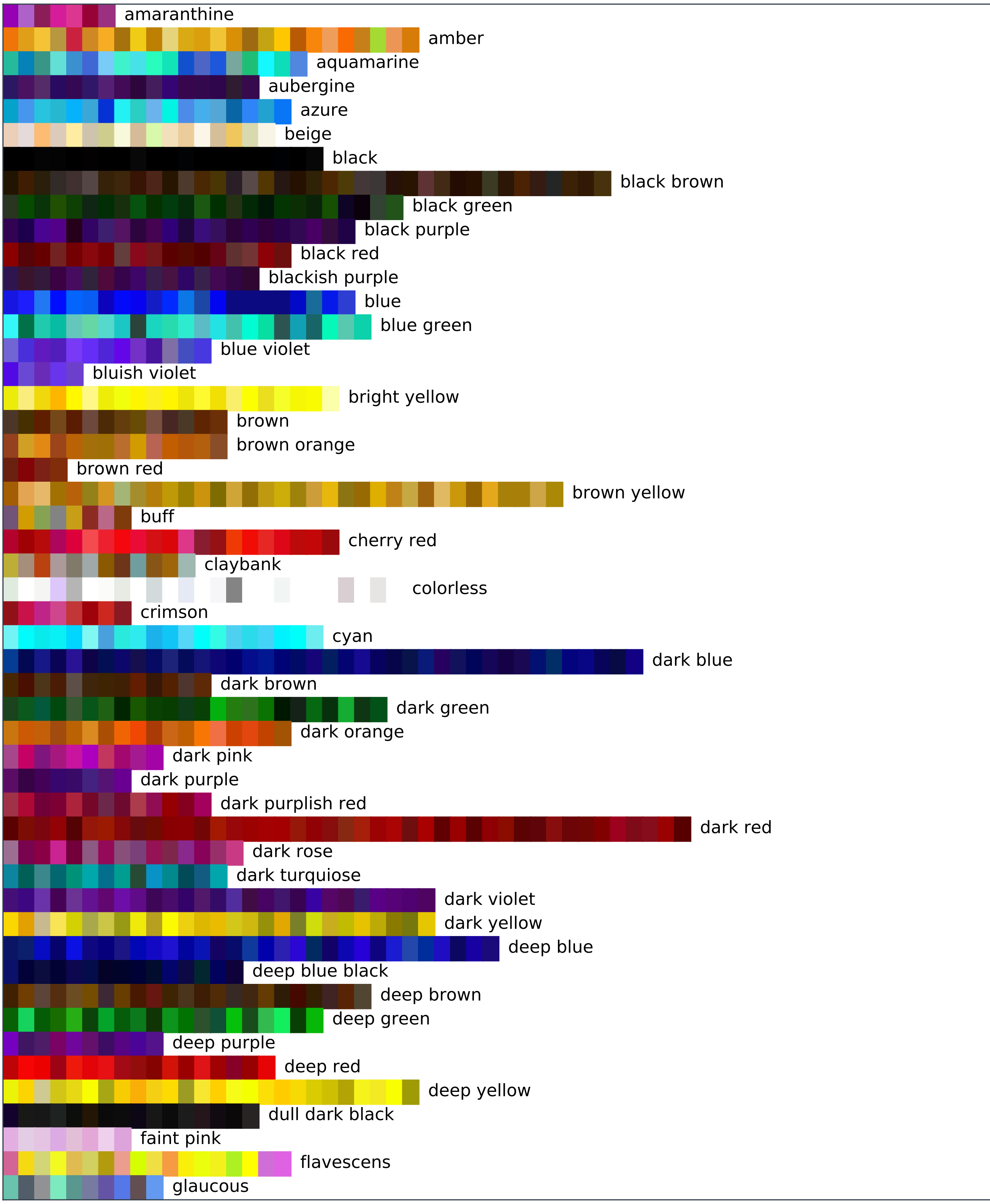

Supplementary Figure 4 | Colours picked by the users in our online survey. After data cleaning. Continued in Supplementary Figures 5 and 6.

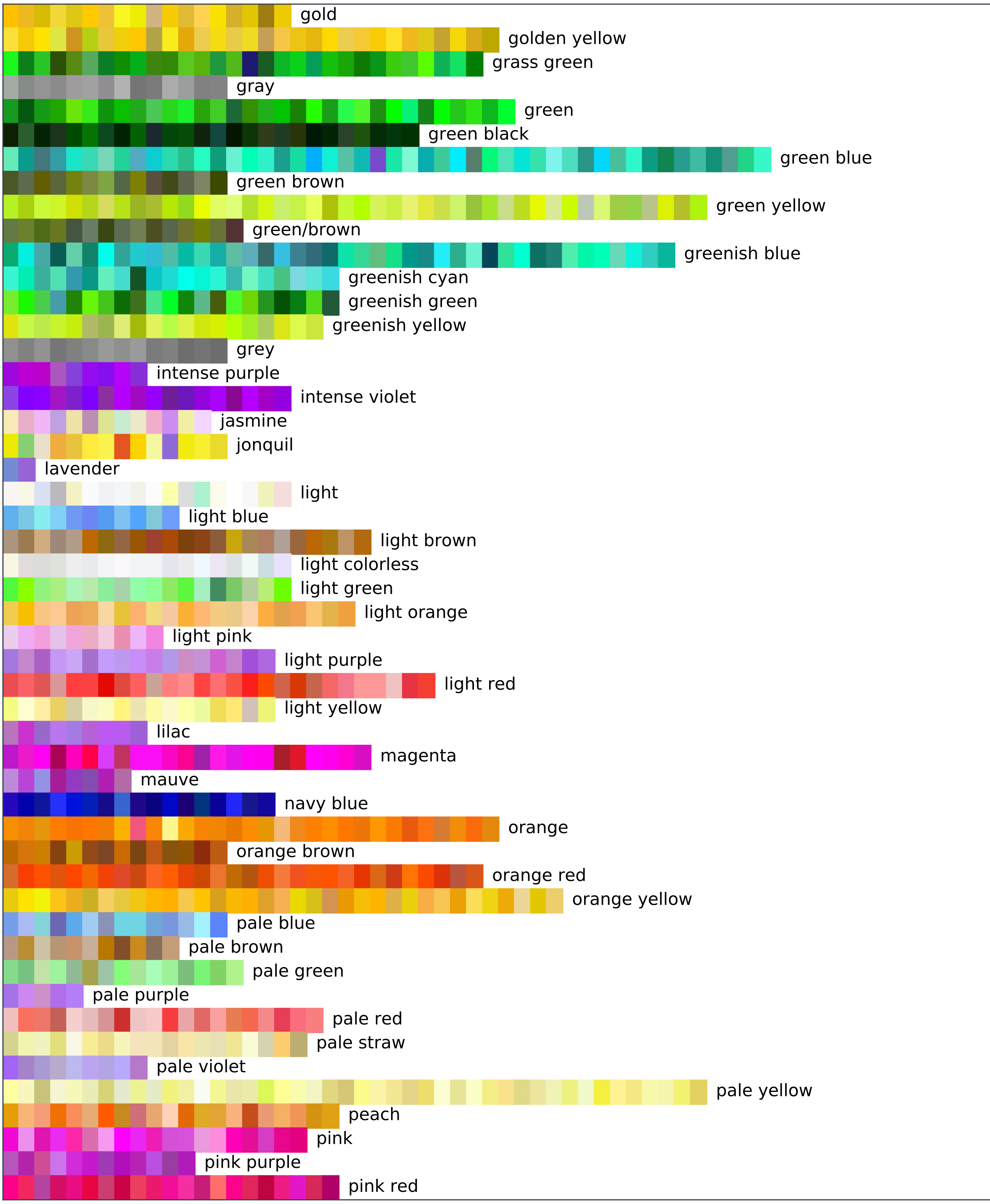

Supplementary Figure 5 | Colours picked by the users in our online survey. After data cleaning. Continued in Supplementary Figure 6.

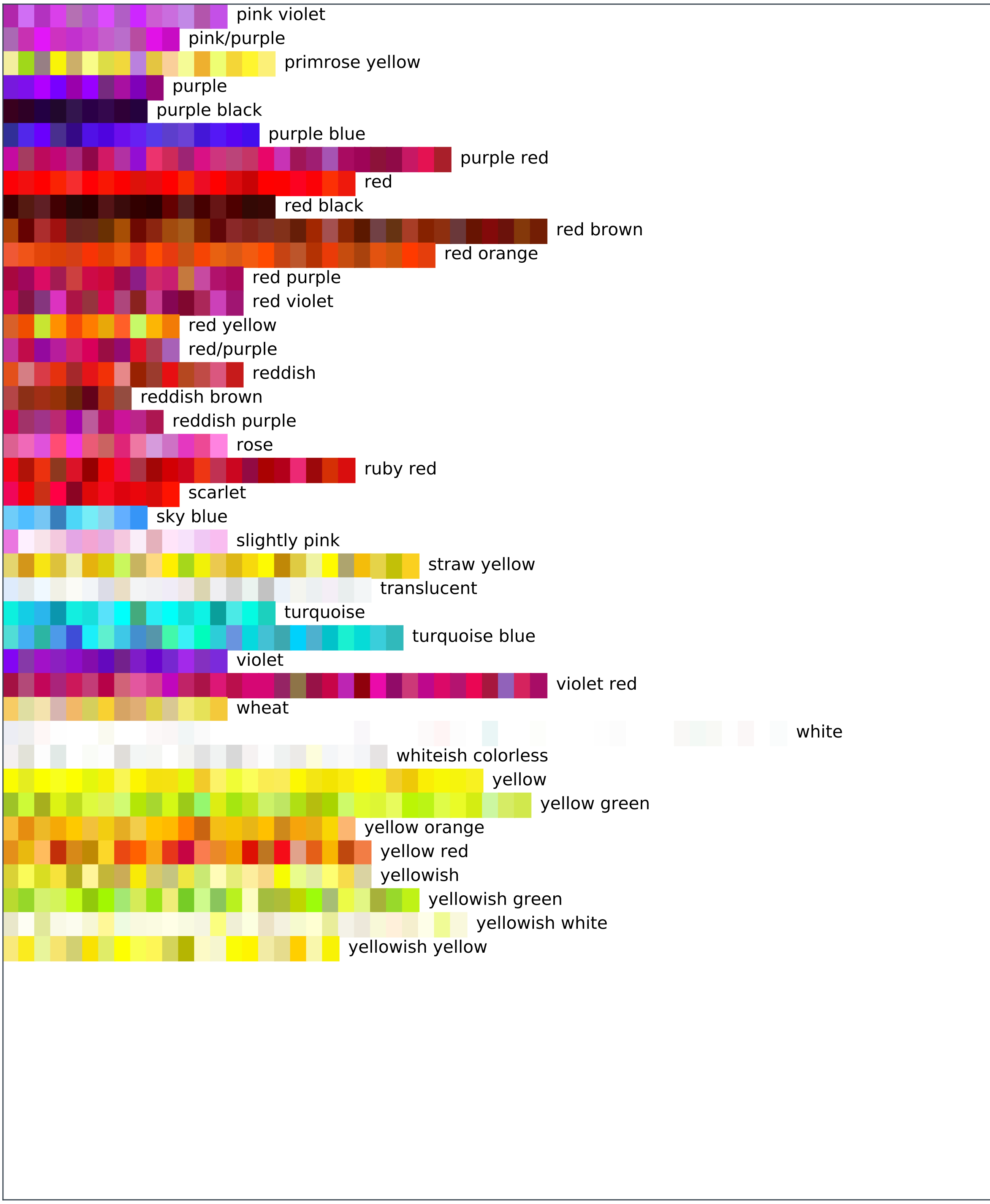

Supplementary Figure 6 | Colours picked by the users in our online survey. After data cleaning.

Another interesting question to ask is whether there is a relationship between the variance and the time the users take to select a colour, which might indicate that there are some colours for which observers are generally more uncertain. The alternative could mean that for some colours there is simply a wide range in perception that is independent of how certain the participants are, i.e., how long they take to pick a colour. Notably, we observe no correlation (cf. Supplementary Figure 7), especially for the unweighted variance.

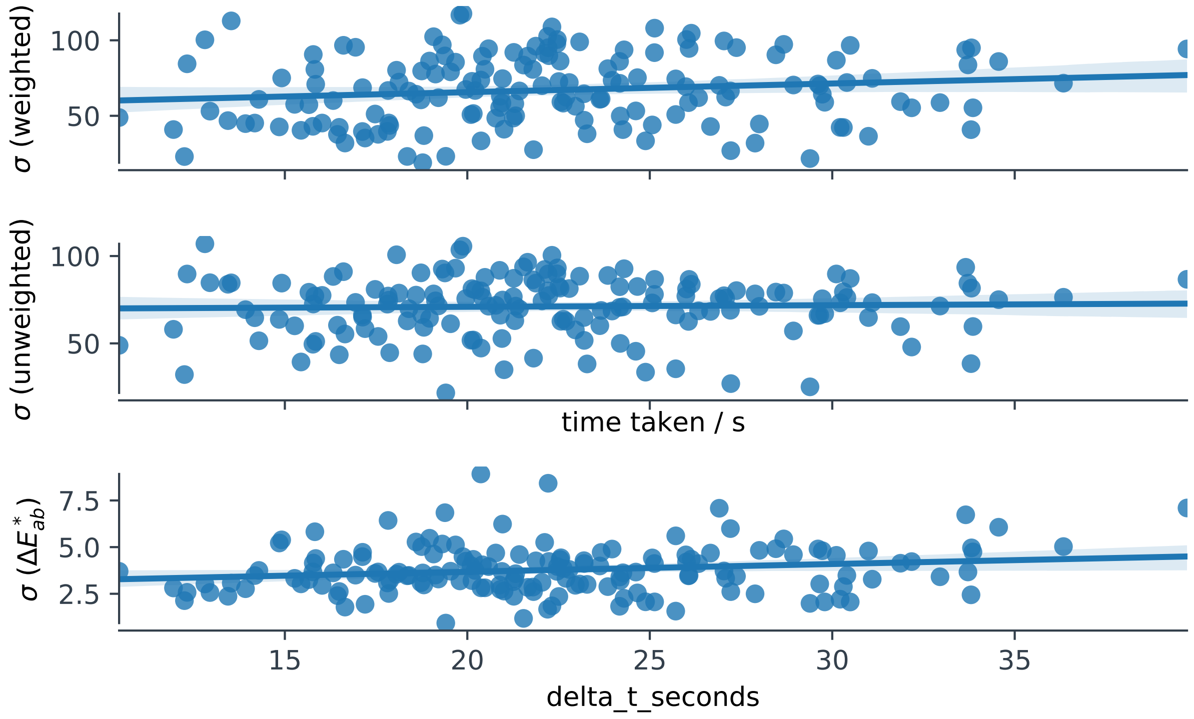

Supplementary Figure 7 | Scatter plots showing plotting the (weighted) standard deviation against the time taken. The deviation for the ΔE^*_{ab} is calculated as $\sqrt{\sum_i \left(\Delta E^*_{ab}(x_i, \mu_{1/2})\right)^2 / N-1}$, where $\Delta E^*_{ab}(x_i, \mu_{1/2})$ is the colour difference between the ith colour and the median. 95 % prediction intervals, as determined using the bootstrap technique, shown as shaded region. Pearson correlation coefficients: weighted 0.14 ($p = 0.08$), unweighted: 0.04, ($p = 0.62$), ΔE^*_{ab} 0.18 ($p = 0.02$). Spearman correlation coefficients: weighted 0.16 ($p = 0.05$), unweighted: 0.04 ($p = 0.62$), ΔE^*_{ab} 0.14 ($p = 0.1$).

The use of our survey data to encode the colour strings is much more useful if our results are representative. A good way to estimate if this is the case is to compare our results with the ones from the xkcd survey, where nearly half a million users were asked the reverse question, i.e., to give a name to a colour which they were shown. The comparison is shown in Supplementary Figure 8: The median colour of our cleaned survey

data is generally close to the result from the xkcd survey. We find a mean $\mu(\Delta E^*_{ab}) = 8.4$ and a median $\mu_{1/2}(\Delta E^*_{ab}) = 6.9$, these values are smaller than the average variance in our survey.

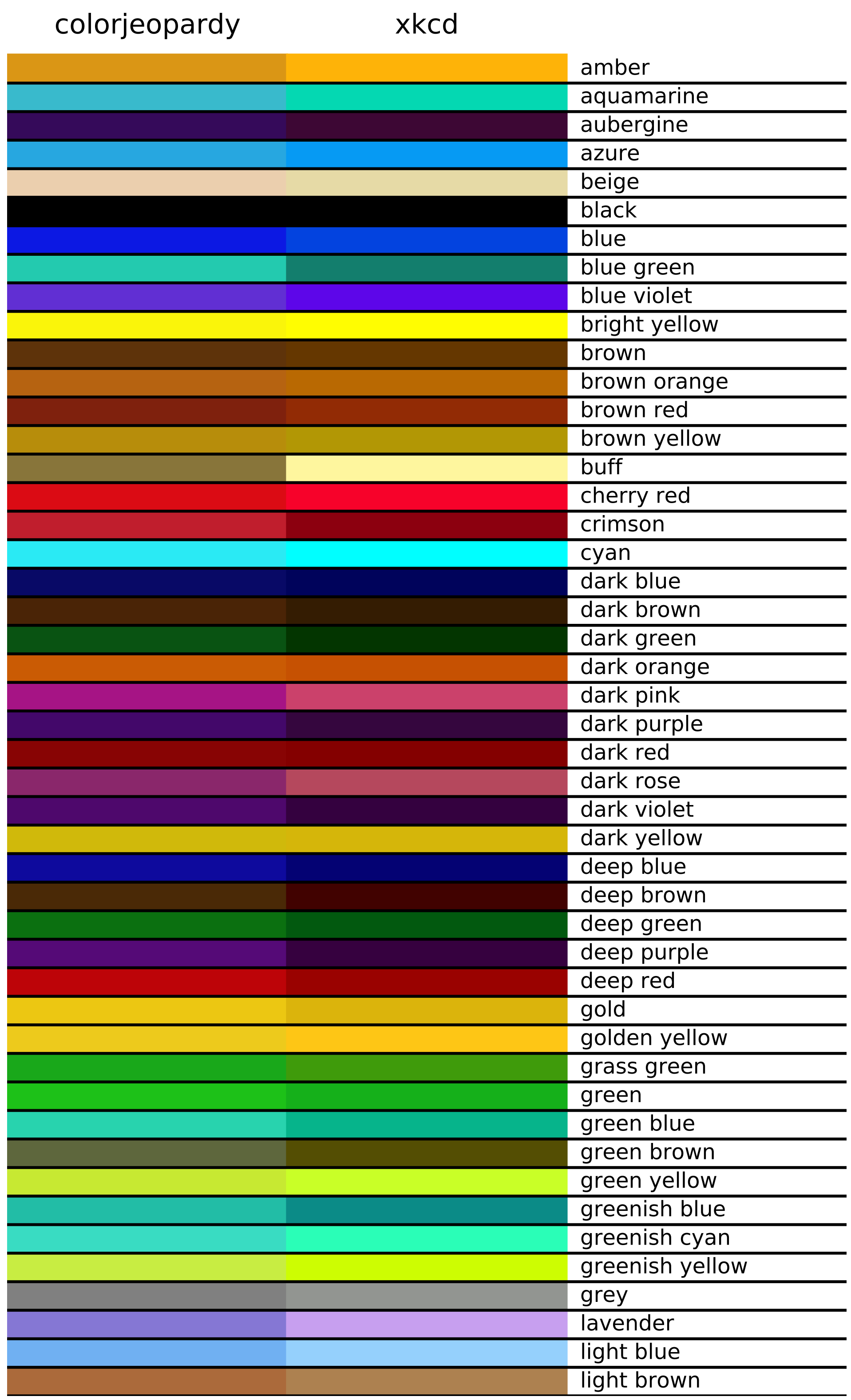

Supplementary Figure 8 | Comparison of colours for which there is an overlap between the xkcd survey and our survey. We aggregated our data using the mean of the cleaned data. Continued in Figure 9.

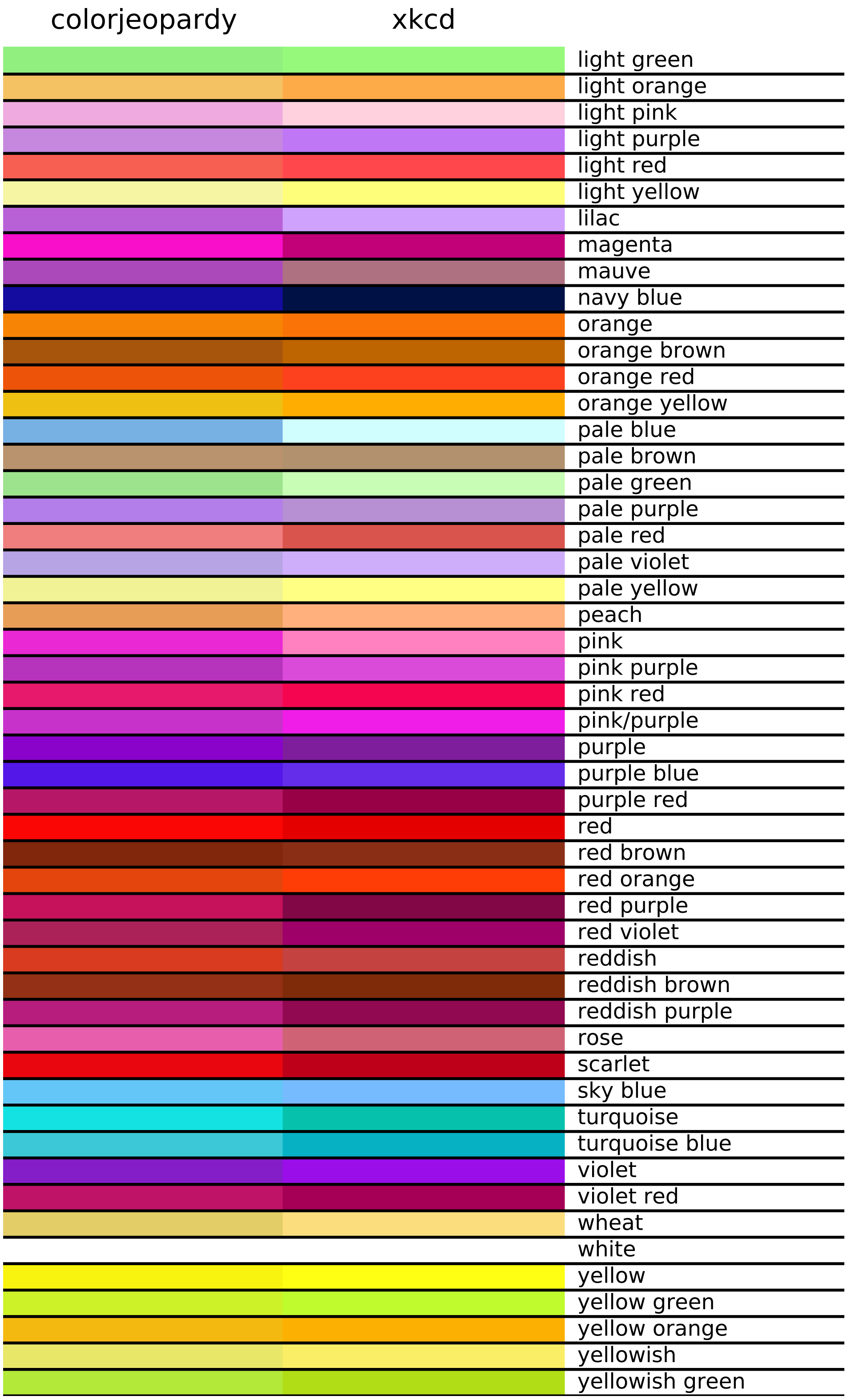

Supplementary Figure 9 | Comparison of colours for which there is an overlap between the xkcd survey and our survey. We aggregated our data using the mean of the cleaned data. Continued from Figure 8.

2 Perceptive spread in colours

Supplementary Figure 10 shows the cumulative distribution of the difference between the colours that were picked in our survey for a given colour string. To reflect that the use of colour strings is not uniform in the Cambridge Structure Database (CSD), we also weight those mean differences by the frequency of the colour string in the metal-organic framework (MOF) subset of the CSD.

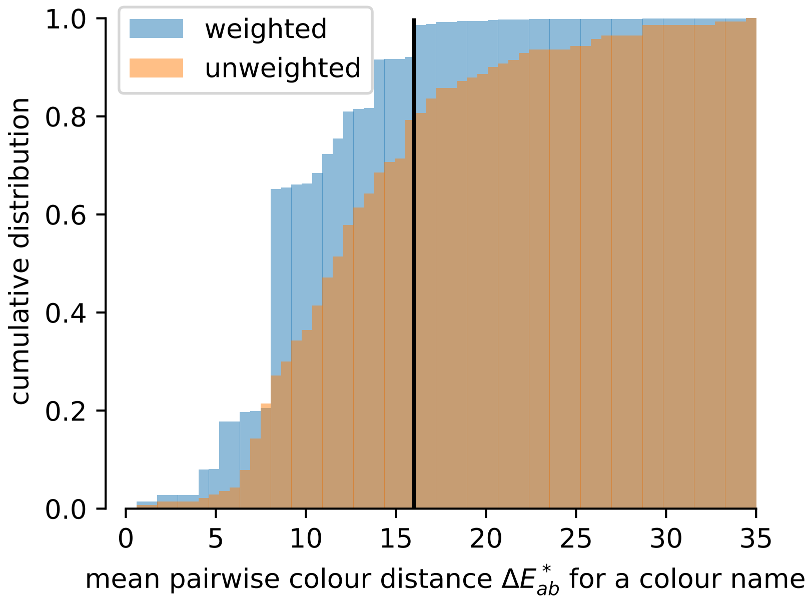

Supplementary Figure 10 | Cumulative distribution of mean pairwise ΔE^*_{ab} for the in survey differences of the colours. In case of the weighted distribution, the data were weighted by the frequency of the colour string in the MOF subset of the CSD.

This plot shows that for most colours the difference in perception is so large that the difference in colours that people pick for the same colour string is so large that it would not comply with common colour reproduction standards—potentially also limiting how well the reproducibility of syntheses can be assessed.

Importantly, this plot also shows that we cannot choose a too tight cutoff on the variance of the colours as we would otherwise too drastically limit the size of our training set. To trade-off the size of the training set and the variance of the perception we chose to only include colours in our training set for which the mean pairwise ΔE^*_{ab} is below 16 (vertical line in Supplementary Figure 10).

3 MOF structures and colour labels

For this study, we used a subset of structures from the Computation-Ready, Experimental (CoRE)-2019 MOF database,[1] for which the colour was deposited in the CSD version 5.4 (November 2019).[2] We used the CSD Python API to retrieve the colour attribute of the CSD entries. We restricted ourselves to structures from the CoRE-MOF database as the code that generates the revised autocorrelation (RAC) assumes that the structures are non-disordered.
For 9525 structures in the CoRE database we could generate RAC fingerprints (we reused the ones we generated for the work by Moosavi et al.[3]). For 8632 of structures in the CoRE database we found a colour in the CSD. After filtering out structures for which featurization failed or the colour label showed a too high variance in our survey we ended up with 6423 structures, from which we dropped 590 duplicates to avoid data leakage and biases.

3.1 Elemental composition of the dataset

The complete dataset, i.e., training, validation and test set combined had the following element counts (counts incremented by one if element present in the structure): C: 5547, H: 5294, O: 4941, N: 3657, Zn: 1074, Cu: 940, Cd: 619, Co: 603, P: 498, S: 415, Mn: 361, Ni: 258, Ag: 248, Eu: 194, Fe: 165, Cl: 162, Tb: 158, F: 147, Gd: 135, La: 125, Nd: 106, Na: 105, Mg: 104, Dy: 100, Sm: 93, Mo: 91, I: 90, In: 90, Er: 86, Ca: 85, U: 85, W: 75, Pr: 66, K: 64, Al: 61, V: 60, Si: 59, Ce: 58, Ga: 55, Ho: 47, Br: 47, Yb: 41, Sr: 40, Cr: 35, Ba: 33, Y: 32, Li: 32, B: 24, Tm: 20, Ru: 19, Pt: 18, Sc: 18, Au: 15, Be: 12, Re: 12, Pd: 10, Lu: 10, Se: 10, Nb: 9, Cs: 8, Hg: 6, Bi: 6, Pb: 6, As: 5, Ge: 5, Th: 5, Hf: 5, Rb: 5, Sb: 4, Sn: 4, Ir: 2, Rh: 1.

4 Model architecture

During the development of the model we experimented with many model architectures going from Bayesian neural network (BNN), over Gaussian process regression (GPR) (with coregionalized kernels) to gradient boosted decision tree (GBDT). Many of our development attempts are tracked on `comet.ml` (`https://www.comet.ml/kjappelbaum/color-ml?shareable=jfE6okDmxlnYimYFFnsJcMCO6`) and `wandb`[4] (`https://app.wandb.ai/kjappelbaum/colorml/`) and the corresponding code is also available on GitHub (`https://github.com/kjappelbaum/colorml`).

Finally, we decided to use the `LightGBM`[5] implementation of GBDT as it implements the quantile loss, which can be used to also predict prediction intervals. By switching the loss function to quantile loss one can use these models to predict the estimated prediction intervals.[6] The quantiles of some conditional distribution $\mathbb{P}(Y|x)$ estimate $f_\alpha(x) = y$ subject to $\mathbb{P}(Y < y|x) = \alpha$ ($\alpha \in (0, 1)$). For example, the 0.5 quantile is just the median and corresponds to minimising the mean absolute error, which is symmetric for over- and underprediction. The loss for other quantiles penalises negative errors more (for higher quantiles) or less (for lower quantiles) than positive errors. In this work, we trained a GBDT to predict the median and the 10th as well as the 90th percentile of the colour channels, which allows us to provide estimated prediction intervals.

4.1 Features

4.1.1 Revised autocorrelation functions (RACs)

Difference RACs are computed as follows

$$^{\text{start}}_{\text{scope}}P_d^{\text{diff}} = \sum_i^{\text{start}} \sum_j^{\text{scope}} (P_i - P_j)\delta(d_{i,j}, d), \tag{1}$$

where, atomic property P of atom i (part of the *start* atom list) is correlated to atom j (part of the *scope* atom list) when they are separated by d number of bonds.

Analogously, product RACs are defined as

$$^{\text{start}}_{\text{scope}}P_d^{\text{diff}} = \sum_i^{\text{start}} \sum_j^{\text{scope}} (P_i P_j)\delta(d_{i,j}, d). \tag{2}$$

For this work we considered a maximum depth of three. More details about the implementation can be found in Moosavi et al.[3]

4.1.2 Additional linker features

Using the SMILES extracted using the MOFid code[7], we computed for the linkers

- number of tertiary amide groups
- number of ester groups
- number of carbonyl groups
- lgP
- molar refractivity
- number of aromatic bond
- number of aromatic rings
- number of double bonds

We considered both the sum and the average of those counts for all linkers in a primitive cell of a MOF as descriptors.

4.2 Hyperparameter optimization

We performed a hyperparameter optimisation over a wide range of parameters using the Bayesian optimiser (using Gaussian processes as surrogate models) and hyperband algorithm implemented in `wandb`.[8] We used the 5-fold cross-validated error as the empirical error estimate.

For efficiency reasons, we the estimators for different colour channels shared hyperparameters. The ranges we considered and the hyperparameters which we used in the final model are listed in Supplementary Table 1.

The influence of the different hyperparameters is shown as a parallel coordinates plot in Supplementary Figure 11.

Supplementary Table 1 | Range of hyperparameters considered and their final values for the models. The names in parentheses indicate the run names on `wandb`.

parameter name	range	final value median (run `laced-sweep-20`)	final value 0.1 (run `vague-sweep-48`)	final value 0.9 (run `cerulean-sweep-157`)
n_estimators	(100, 5000)	4983	454	4813
max_depth	(5, 100)	99	20	100
num_leaves	(5, 500)	448	417	256
reg_alpha	(0.00001, 0.4)	1.432	1.356	1.046
reg_lambda	(0.00001, 0.4)	1.04	1.17	1.343
subsample	(0.01, 1.0)	0.3613	0.1287	0.8991
colsample_bytree	(0.01, 1.0)	0.9262	0.7345	0.3986
min_child_weight	(0.001, 0.1)	0.08181	0.01801	0.0484

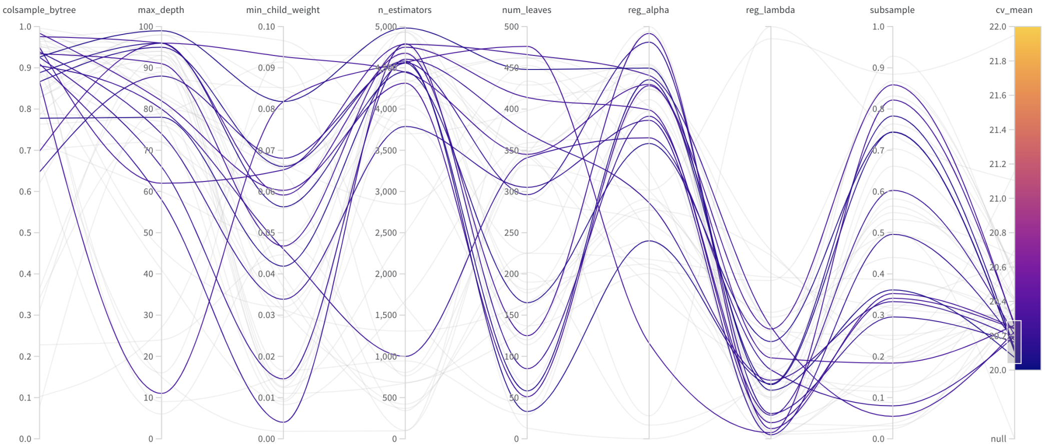

Supplementary Figure 11 | Parallel coordinates plot showing the cross-validated score as a function of the hyperparameter settings for the median model, highlighted are the settings for the best performing models.

4.3 Colourspaces

There are different spaces in which one can define colours, between which one can convert with (non)-linear transformations. The most commonly used colourspace is the sRGB colourspace, but it is typically found that other colour spaces such as Hue, Saturation and Luminance (HSL) can give better performance in some applications. Generally, one can distinguish

between human- (e.g., HSL), hardware- (e.g., RGB) and instrument-oriented (e.g., CIE 1976 L*a*b* (CIELAB)) colour spaces.[9;10] In initial experiences, we varied the colourspace between RGB, HSL and CIELAB. We performed the transformation between the different colour spaces using the `Colour` Python package.[11] Typically, we found the RGB colourspace to perform best, wherefore we used it in the final model.

5 Sensitivity to the cutoff in the variance in perceptive spread and using all datapoints from the survey as noisy labels

Following the analysis of Supplementary Figure 10 we investigated how the predictive performance of our model depends on the cutoff we choose for the variance in the perception of the colours (Supplementary Figure 12, i.e., the mean pairwise ΔE^*_{ab} between the colours picked by the participants of our survey).
To allow for a fair comparison, we first split of a holdout set of the full database (after the preprocessing steps described in section 1) and then applied the threshold on the perceptive variance on the training set. We considered a threshold of 5, 16, as well as no threshold at all on the mean pairwise ΔE^*_{ab} between the survey responses for any given colour.

Moreover, one can imagine that one could use all the data from the survey in such a way that the same MOF is presented to the model multiple times with the different colours our survey participants picked for a given colour string. This is, one MOF feature vector would be mapped to multiple RGB values during training. Those values might be quite similar (for the low threshold on the in-survey variance) or dissimilar (if we do not apply a threshold). This is similar to the addition of noise to labels that is sometimes used to reduce overfitting. To understand the influence of this effect, we trained our model only on the medians of the survey results (one MOF mapped to one RGB value during training) or on all colour labels (one MOF mapped to one RGB value during training) with 50 different train/test splits and measured the ΔE^*_{ab} on the two different test sets. For efficiency reasons (and also following the low sensitivity on the hyperparameter settings that is evident from Supplementary Figure 11) we chose the same hyperparameters for all training sets.

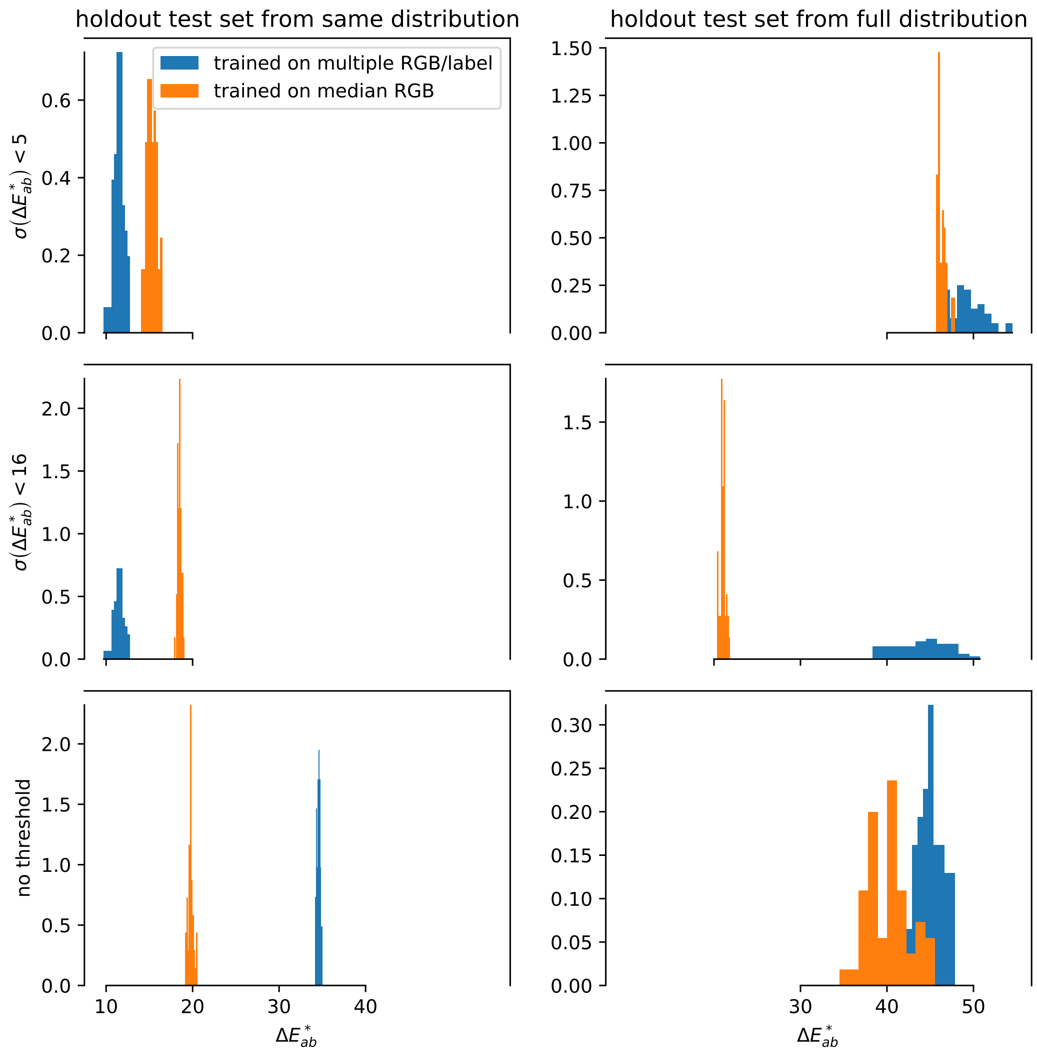

Supplementary Figure 12 | Effect of the variance in the training data on the predictive performance of the model. The histograms show the test errors of 50 independent runs, with new train/test splits. The left column shows the predictive performance on a holdout set which only includes colours with a perceptive variance lower than the threshold. The right column is a constant holdout test set that includes all colours (i.e., we applied no threshold on the variance in the perception). The colours indicate if the model was trained on the means of the survey responses or on all data points. In latter case, one MOF would be mapped to multiple RGB values. The rows show the experiments for different thresholds on the mean pairwise ΔE^*_{ab} of the colours picked in the survey.

We observe that if we apply a threshold, the use of all responses in the survey (i.e., showing the same structure with different RGB values to the model) tends to lead to overfitting (as expected, we observe the best performance on the test set drawn from the same distribution) and poor transfer. If we train on all colours, this approach also leads to poor generalisation on a test set drawn from the same distribution. Analysing the results we

get from training only on the median, a threshold of 16 seems to be least prone to overfitting. It is easy to understand why the tight threshold of 5 performs badly: We drastically limit the number of training points and the chemical space our model sees during training. In contrast, for the case without threshold, there are more responses with a high variance that are prone to be wrong and the data is more difficult to learn for the model.

For these reasons we trained our models on the medians of colours with a in-survey mean pairwise colour distance of $\Delta E^*_{ab} < 16$.

6 Performance measurement

6.1 Representative predictions

The predictions for 100 random structures of the test and train set are shown in Supplementary Figure 13 and Supplementary Figure 14.

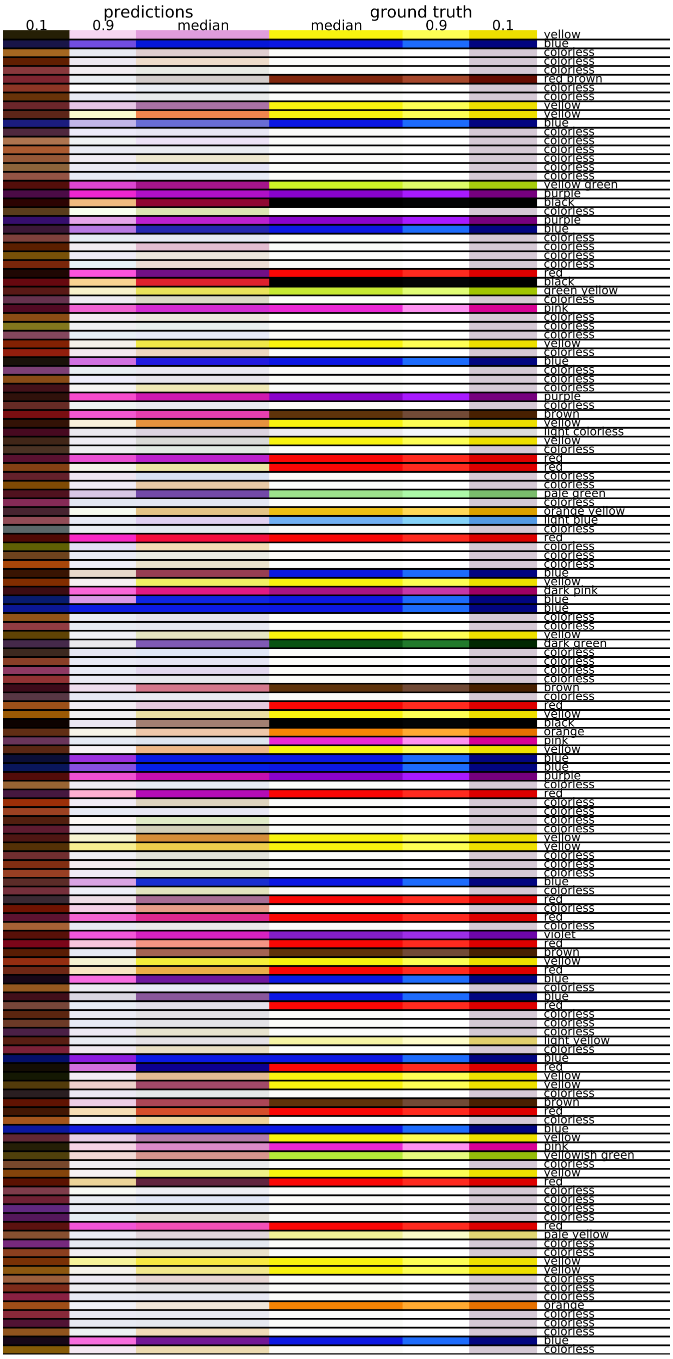

Supplementary Figure 13 | Random 100 predictions of our model on a holdout test set.

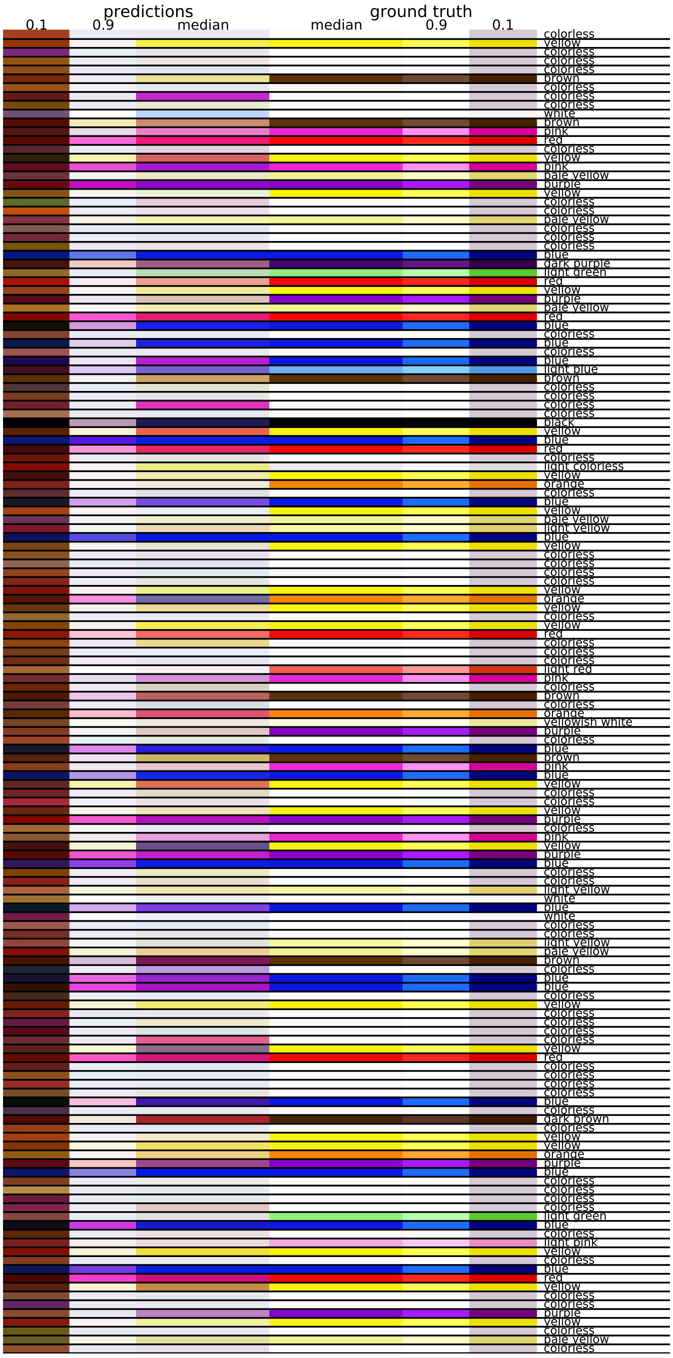

Supplementary Figure 14 | Random 100 predictions of our model on the training set.

6.2 Test in experimental compounds

To test our model we took photos from some structures of our experimental colleagues and excluded structures which descriptor are closer (Manhattan, $p = 1$, distance) than 0.02 to one of the test structures from our training set. All structures except Sion-17 have been prepared according to published procedures. Sion-17 is a MOF that has not been reported so far. We chose to include it in our test set due to the rather unusual chemistry with a Pd porphyrin.

For UiO-66-NH_2(Nb) we used the samples prepared the work reported by Syzgantseva et al.[12]

6.2.1 UiO−66−NH_2

UiO-66-NH_2 was synthesised by a method adapted from Cavka et al.[13] Briefly, stock solutions of the zirconium precursor (320 mg of zirconium(IV)chloride ($ZrCl_4$ Aldrich), sonicated in 20 mL of DMF) and ligand (240 mg of 2-aminoterphthalic acid (H_2BDC-NH_2, Aldrich), dissolved in 20 mL DMF) were prepared. 2 mL of each solution was pipetted into 10 × 12 mL glass reactor vials. The reactor vials were heated to 120 °C for 48 hours, then cooled at a rate of 0.2 °C min^{-1} to room temperature. The resulting pale-yellow material was combined and washed by centrifuge x 3 with DMF (40 mL), and x 2 with methanol (40 mL), and left overnight to dry at room temperature

6.2.2 UiO-NDC

UiO-66-NDC was synthesised by a method adapted from Cavka et al.[13] 25 mg of of zirconium(IV)chloride ($ZrCl_4$, Aldrich), and 25 mg of 1,4-Napthalene-dicarboxylic acid (H_2NDC, Aldrich) was added to a 12 mL reactor vial containing 4 mL of DMF. The solution was sonicated for 5 minutes, to afford a clear yellow tinged solution, and then 0.1 mL of acetic acid was added. The vial was then sealed, and heated to 120 °C for 48 hours, then cooled at a rate of 0.2 °C min^{-1} to room temperature. The resulting material was washed by filtration by 3 x 10 mL of DMF, and 1 x 10 mL of acetone, and left overnight to dry at room temperature.

6.2.3 Mg-MOF-74

Mg-MOF-74 was synthesised by a method adapted from Millward and Yaghi.[14] 125 mg of magnesium(II)nitrate hexahydrate ($Mg(NO_3)_2.6H_2O$, Aldrich) and 100 mg of 2,5-dihydroxyterephthalic acid (H_2HBSC) was added

to a 25 mL glass reactor containing 10 mL of DMF. After sonicating for 10 min, 0.5 mL of 1 propanol, and 0.5 mL of water was added to the solution. The reactor was sealed, and then heated to 100 °C for 20 hours, and then cooled at a rate of 0.1 °C min^{-1} to room temperature. The resulting crystals were washed with DMF by syphoning off the mother liquor with a pipette, and replacing with 10 mL of DMF. After gently shaking the vial, the DMF was again syphoned off and replaced with fresh DMF. This procedure was repeated 5 times. The crystals were then filtered, and allowed to dry overnight on the filter paper at room temperature.

6.2.4 Co-MOF-74

A mixture of cobalt(II)nitrate hexahydrate (970 mg, 4.61 mmol), 2,5-dihydroxyterephthalic acid (198 mg, 999 µmol), ethanol (27 mL), N,N-dimethylformamide (27 mL, 349 mmol, 1 eq.), and water (27 mL, 1.5 mol, 4.29 eq.) was transferred into a 250 mL Pyrex jar. The jar was placed for 10 min in an ultrasonic bath until a transparent solution was reached. The glass jar was sealed and kept at 100 °C in an oven for 24 h. After that, the dark red crystals were filtered and washed with ethanol three times. Then, the product was allowed to be air dried.

6.2.5 Zn-MOF-74

A mixture of 2,5-dihydroxyterephthalic acid (240 mg, 1.21 mmol), zinc nitrate hexahydrate (720 mg, 2.42 mmol), water (3 mL) and N,N-dimethylformamide (27 mL) were transferred into a 100 mL pyrex jar. The jar was placed for 10 minutes in an ultrasonic bath until a transparent solution was reached. The glass jar then was sealed and kept at 120 °C in an oven for 3 days. After that, the yellow crystals were filtered and washed with ethanol three times. Then, the product was allowed to be air dried.

6.2.6 Sion-17 (first reported in this work)

10 mg of Pd(II) meso-Tetra(4-carboxyphenyl)porphine (H_4-TpCPP-Pd, Frontier Scientific) was placed into a 12 mL glass reactor, in addition to 5 mg of Zinc(II)nitrate hexahydrate ($Zn(NO_3)_2.6H_2O$), and 1 mg of adenine ($C_5H_5N^5$, Alfa Aesar) to act as a mediator. To this, 2.5 mL of DMF was added, and the solution was sonicated for 10 min. After sonication, 0.5 mL of water was added, and then the solution was acidified with 400 µL of 1M nitric acid. The reactor was then sealed and heated to 120 °C for 72 hours, then cooled to room temperature at a rate of 0.2 °C min^{-1}. The resulting crystals were

washed with DMF by syphoning off the mother liquor with a pipette, and replacing with 5 mL of DMF. After gently shaking the vial, the DMF was again syphoned off and replaced with fresh DMF. This procedure was repeated 5 times. The crystals were then filtered and allowed to dry overnight on the filter paper at room temperature.

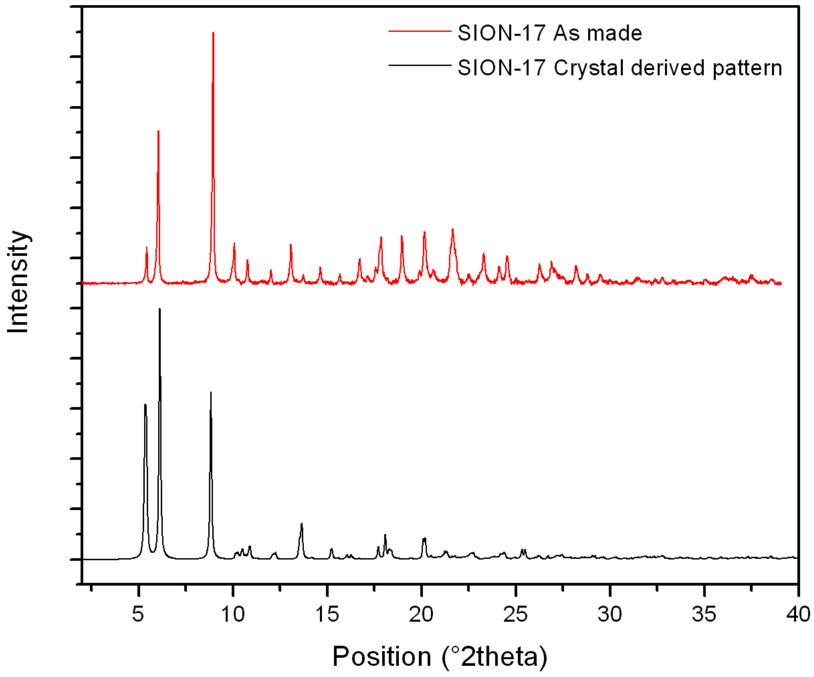

Supplementary Figure 15 | PXRD for as synthesised Sion-17.

6.2.7 HKUST-1

1,3,5-benzenetricarboxylic acid (210 mg, 999 µmol) and copper(II)nitrate hemi(pentahydrate) (348 mg, 1.5 mmol) were added to a 12 mL microwave vial. To this, N,N-dimethylformamide (2.5 mL), ethanol (2 mL) and finally water (500 µL) were added. The vial was then capped and crimped, and sonicated until the material dissolved (approx. 2 min). A clear, pale blue solution was formed.

The vial was placed in a microwave, and heated at a power of 200 W to 140 °C for 20 min. The microwave was then cooled for 8 min to 40 °C.

A blue powder was formed. The material was washed by centrifugation (5 x 30 mL N,N-dimethylformamide) in a 50 mL size centrifuge tube at 4000 rpm for 5 min. The solvent was removed from the centrifuge tube, and

the material was then allowed to dry in the centrifugation tube at room temperature over 2 days.

Approximately 100 mg of material was activated for 24 h at 200 °C under vacuum in a Schlenk tube. The blue colour darkened.

6.2.8 Cu-TDPAT

2,4,6-tris(3,5-dicarboxylphenylamino)-1,3,5-triazine (422 mg, 682 µmol), copper(II)nitrate trihydrate (2.31 g, 11 mmol), N,N-dimethylacetamide (30 mL), 1,4-dioxane (30 mL), water (1.5 mL), and fluoroboric acid (13.5 mL) were mixed and stirred for 5 minutes in a 250 mL jar. Then, the solution was located inside the 85 °C oven for 3 days. Then it was taken out of the oven. After decanting the hot mother liquor, the crystals were rinsed with DMAc and methanol and dried. In order to activate the sample, firstly, the solvent exchange process should be done. In order to do this and also to ensure the extraction of any unreacted ligand, the material was inserted into a thimble and washed extensively with methanol in a soxhlet apparatus overnight. The solvent-exchanged sample then was activated by heating at 150 °C for 12 hours under vacuum. The sealed activated sample then was transferred into the glove box.

6.2.9 AlPMOF

Al-PMOF was synthesised by a method adapted from Fateeva et al.[15] 100 mg of meso-Tetra (4-carboxyphenyl)porphine (H_4-TpCPP-H_2, Frontier Scientific), and 60 mg of aluminium(III)chloride hexahydrate ($AlCl_3.6H_20$) were added to a 23 mL PTFE reactor. To this, 8 mL of milipore water was added, and 2 mL of DMF. The reactor was placed into a Parr Vessel, and heated to 180 °C for 16 hours, then cooled to room temperature at a rate of 1.5 °C min^{-1}. The product was washed by centrifuge x 5 with DMF, and x 2 with acetone, and left to dry overnight at room temperature.

6.3 Examples of errors

In the following, we discuss some cases where our prediction is distant ($\Delta E^*_{ab} > 30$) from the colour reported in the CSD and for which we found a report of the colour in the original journal article.
We see that in some instances the colour deposited in the CSD is not the same as the one reported in the original reference. Of course, sometimes our model is also simply wrong. Values in parentheses give the RGB coordinates, names are typically the closest HTML colour names.

- `SOQHIR01` is deposited in the CSD as orange, the paper reports colourless rod-like crystals.[16] We predict old lace (240, 232, 218).
- `IZOWEA` is reported as red in the CSD and as dark red in the paper.[17] We predict a burgundy colour (99, 36, 64) which closest HTML name pale violet red.
- `YORLEY` is reported in the CSD as yellowish green crystals that turn orange within a week.[18] We predict a rosy brown (215, 150, 143).
- `PUPYAA` is reported in the CSD and the paper[19] as yellow. We predict plum (181, 123, 170).
- `PULDOQ` is reported in the CSD as yellow and as blue block crystals in the paper.[20] We predict a pale violet red (162, 73, 105).
- `GAMXAV` is reported in the CSD and the paper[21] as red. We predict a dark blue colour (14, 0, 144).
- `NEGMUI` is reported in the CSD and the paper[22] as red. We predict a peru color (235, 175, 72).
- `FIFMIS` is reported in the CSD and the paper[23] as red. We predict a medium violet red (223, 41, 143).
- `AVUCEA` is deposited in the CSD as red and also described as such in the paper[24]. We predict a medium violet red (204, 34, 192).
- `GIVHIC` is reported in the CSD and the paper[25] as red. We predict a plum color (169, 110, 148).
- `MAGBUT01` is reported in the CSD and the paper[26] as yellow. We predict a peru color (214, 142, 58).
- `POHWIU` is reported as red in the CSD and as dark red in the paper.[27] We predict a dark magenta (180, 12, 182).

- `FOLLEZ` is reported as pink in the CSD and as colorless in the paper.[28] We predict alice blue (224, 230, 240).
- `GARMAQ` is reported in the CSD as black and as brown in the paper[29]. We predict rosy brown (164, 127, 114).
- `DOZCEC` is reported in the CSD as brown and in the paper as brownish-red[30]. We predict pale violet red (213, 120, 140).
- `WOPWOO` is deposited in the CSD as dark red and as dark violet in the paper.[31] We predict a light pink (216, 147, 178).
- `YICGOI` is deposited as orange in the CSD and reported as such in the paper.[32] We predict a medium violet red (218, 36, 147).

6.4 Baseline models

Before building the GBDT models we evaluated the performance of some simple baseline models. This is important to understand what the minimum performance one can expect. Some metrics are summarised in Supplementary Table 2 and examples of the predictions on a test set are shown in Supplementary Figures 16 and 16.

Supplementary Table 2 | Errors computed in normalised colour space and aggregated with uniform average.

model	r^2 score	MAE	MSE	mean ΔE^*_{ab}
mean baseline	0.00	0.31	0.13	30.2
median baseline	-0.31	0.24	0.17	28.6
$k$nn (k=1)	-0.03	0.19	0.14	20.8
$k$nn (k=4)	0.18	0.22	0.11	24.9

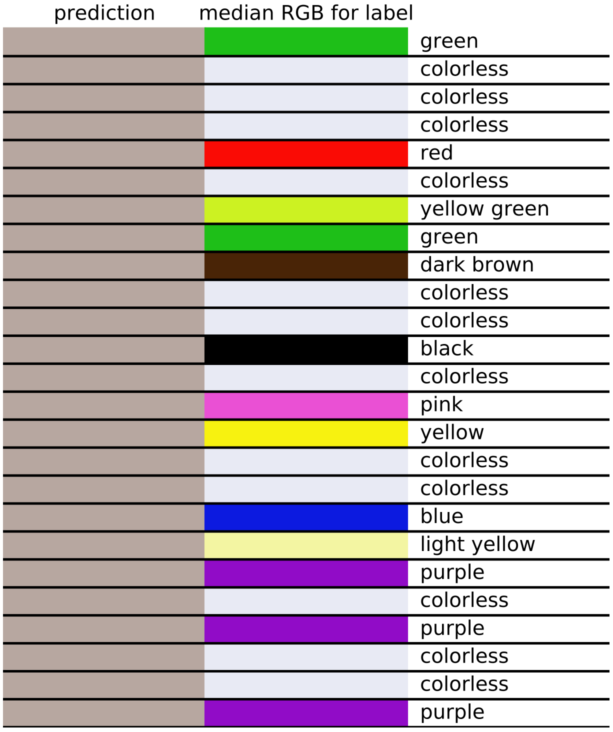

Supplementary Figure 16 | Predictions of a mean baseline on random test samples.

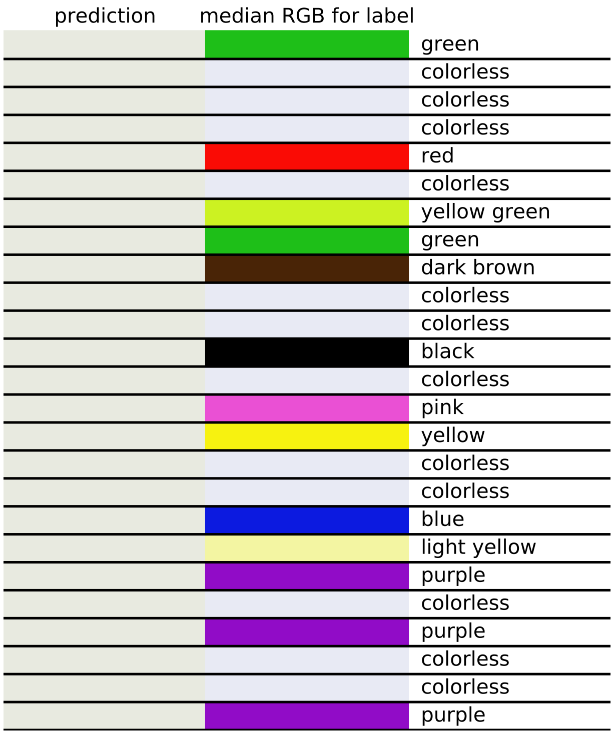

Supplementary Figure 17 | Predictions of a median baseline on random test samples.

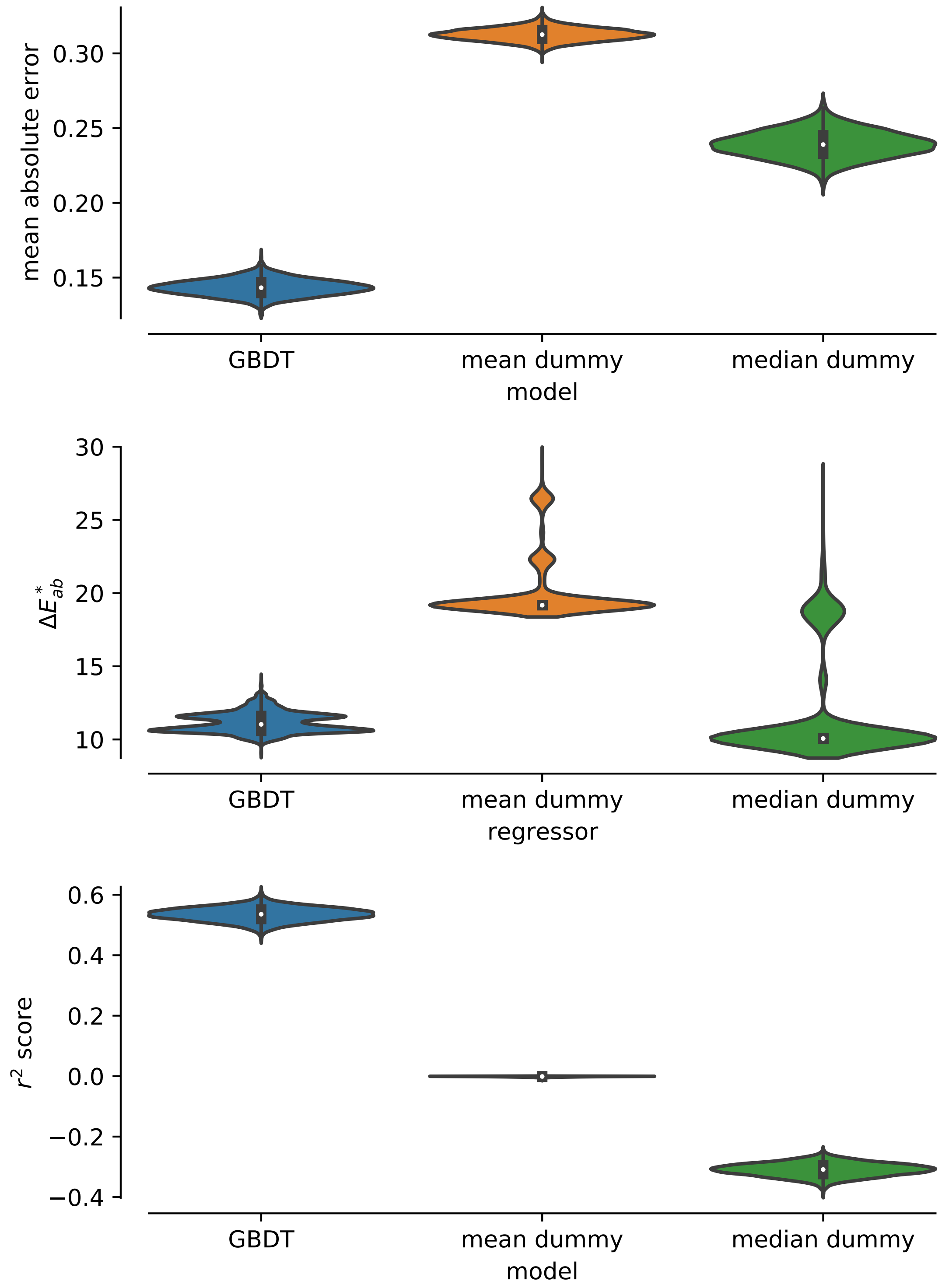

Supplementary Figure 18 | Comparison of the performance of dummy models with the one of our GBDT model. Violinplot shows the distribution of metrics for 5000 bootstrap samples of the test set.

6.5 Permutation test

One method to assess whether the model learned a meaningful association between features and target is to perform a permutation test.[33] For efficiency reasons, we performed this test with only 500 training points and 100 permutations, using the `permutation_test_score` function in `sklearn` (see Supplementary Figure 19).

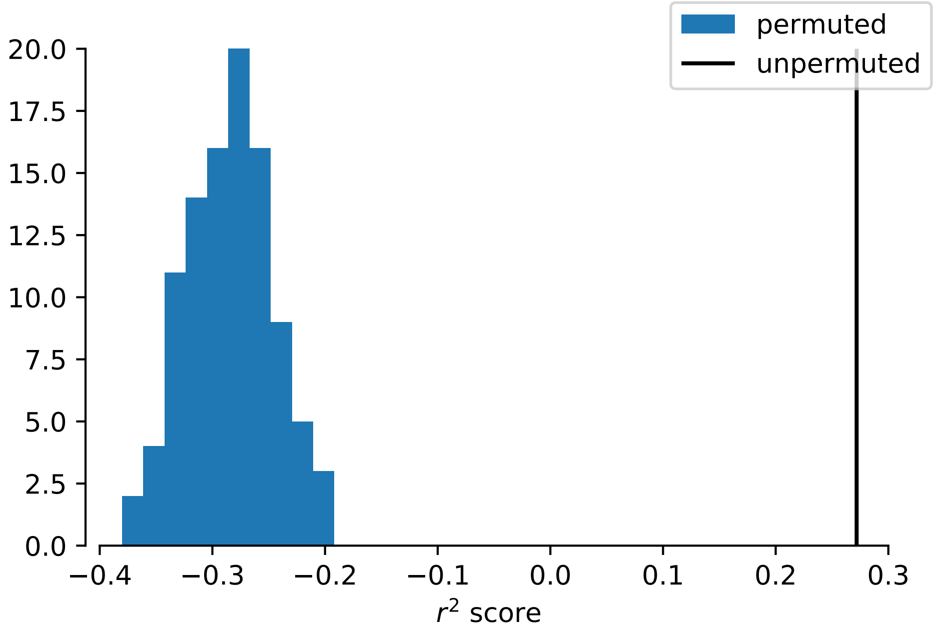

Supplementary Figure 19 | Result of a permutation test ($p = 0.0099$).

6.6 Learning curve

Learning curves for the GBDT model are shown in Figure 20. We observe that the curves did not saturate, i.e., that we could improve our model if we would have more data and that our representation allows the model to learn. (For example, for a non-unique representation one would expect a flat learning curve.[34])

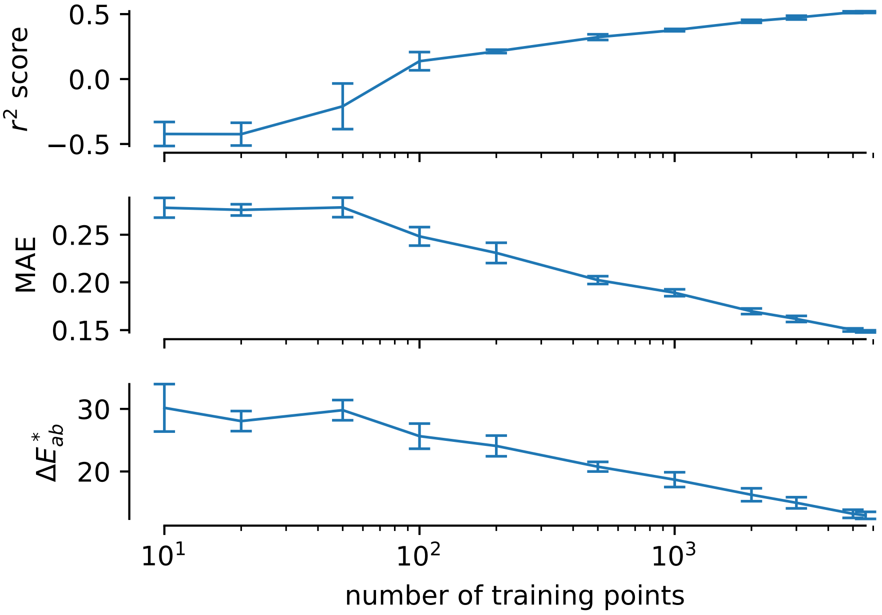

Supplementary Figure 20 | Learning curves for the GBDT trained with 0.5 quantile loss for the r^2 score, the MAE, and the ΔE^*_{ab}. For the learning curves, we sampled ten different training sets, trained the models, and tested on a test set, and show the standard deviation between the ten runs as errorbars.

7 Feature importance analysis

For the blue colour channel, we find high importance (higher than, e.g., the number of double bonds or aromatic rings) of the octanol/water partition coefficient lg P of the linker, which is typically used to quantify hydrophobicity/lipophilicity. To understand the importance of the features better it can be instructive to analyse some ligands in our dataset that minimise or maximise the features. Obviously, just using lg P alone is not predictive—but it can help our model to make good first splits and then refine those based on other features (cf. Figure 21).

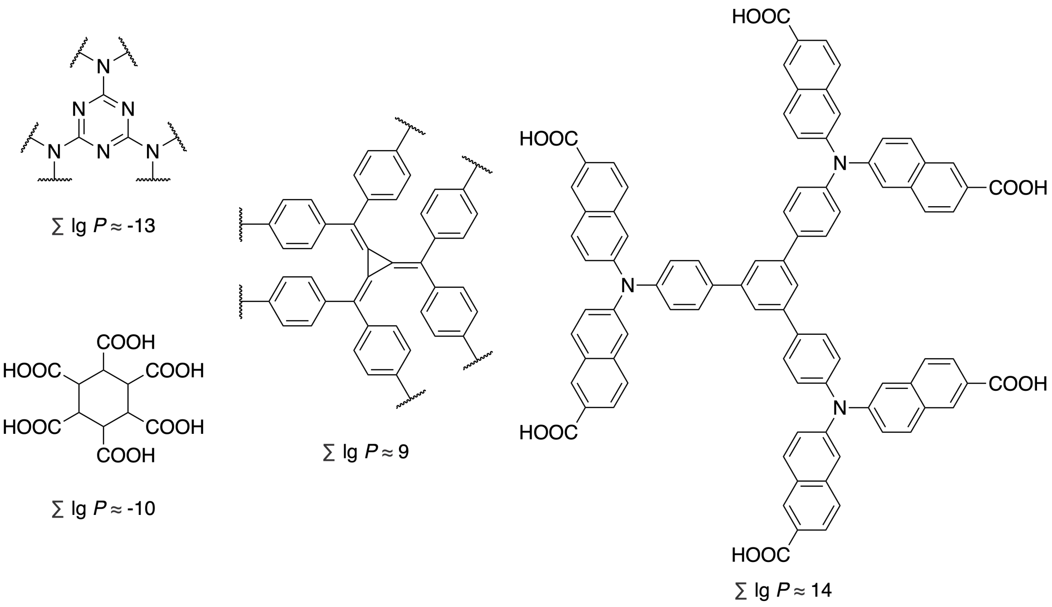

Supplementary Figure 21 | Some examples of ligands that minimise and maximise lg P. The numbers in the ligand report the sum of the lg P across all unique ligands in the MOFs.

7.1 SHAP interaction values

One feat of the SHAP analysis is that it can provide insights into feature interactions. In Supplementary Figures 22–24 we show the strongest absolute interaction values. We can see that the metal-ligand interactions are strongest for the blue colour channel but play a role for every colour channel. Interaction values are shown for a random subset of 1000 structures from the training set.

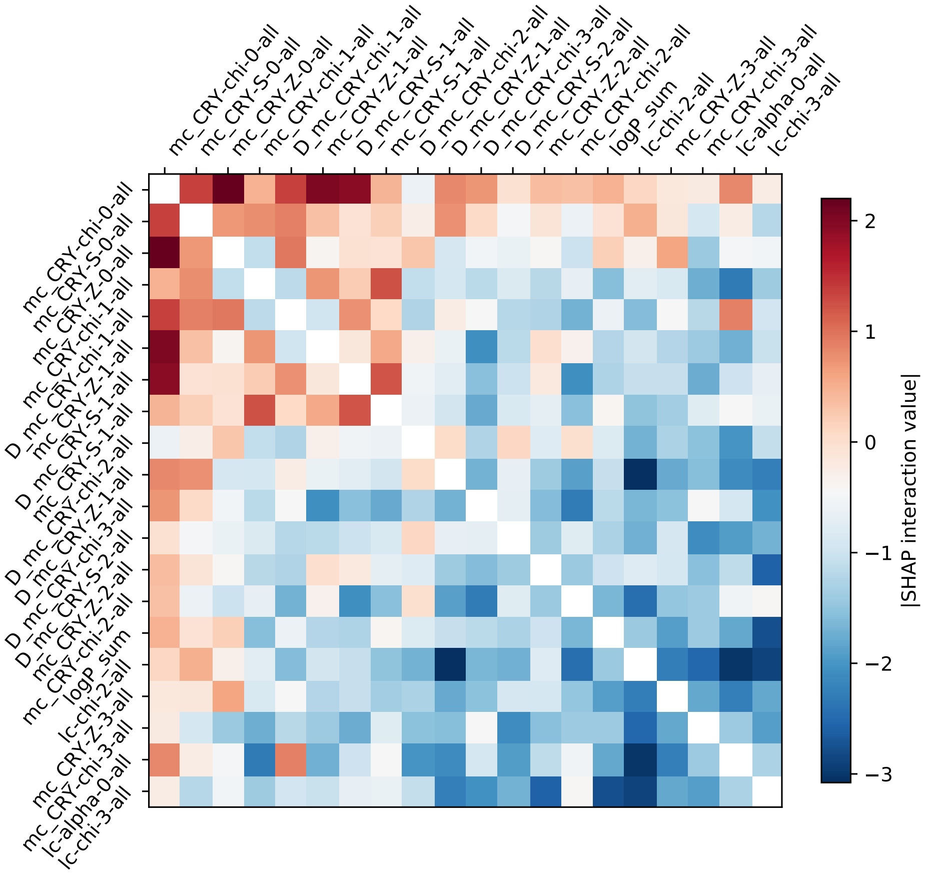

Supplementary Figure 22 | Highest absolute SHAP interaction values for the red colour channel.

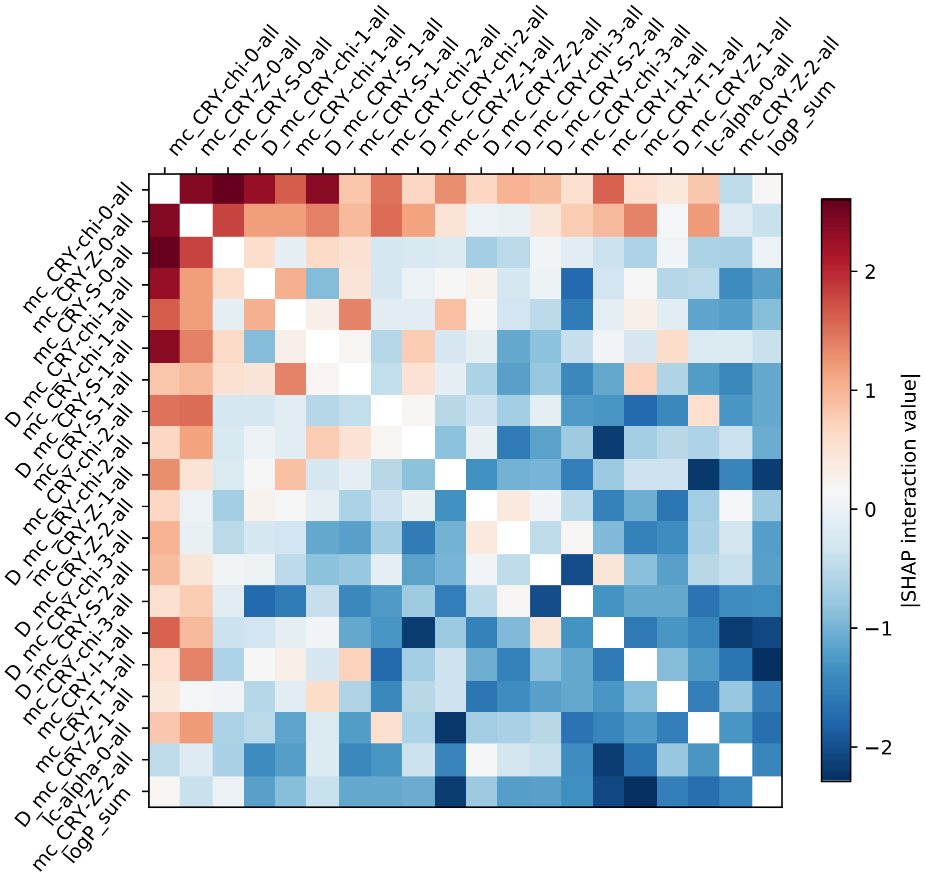

Supplementary Figure 23 | Highest absolute SHAP interaction values for the green colour channel.

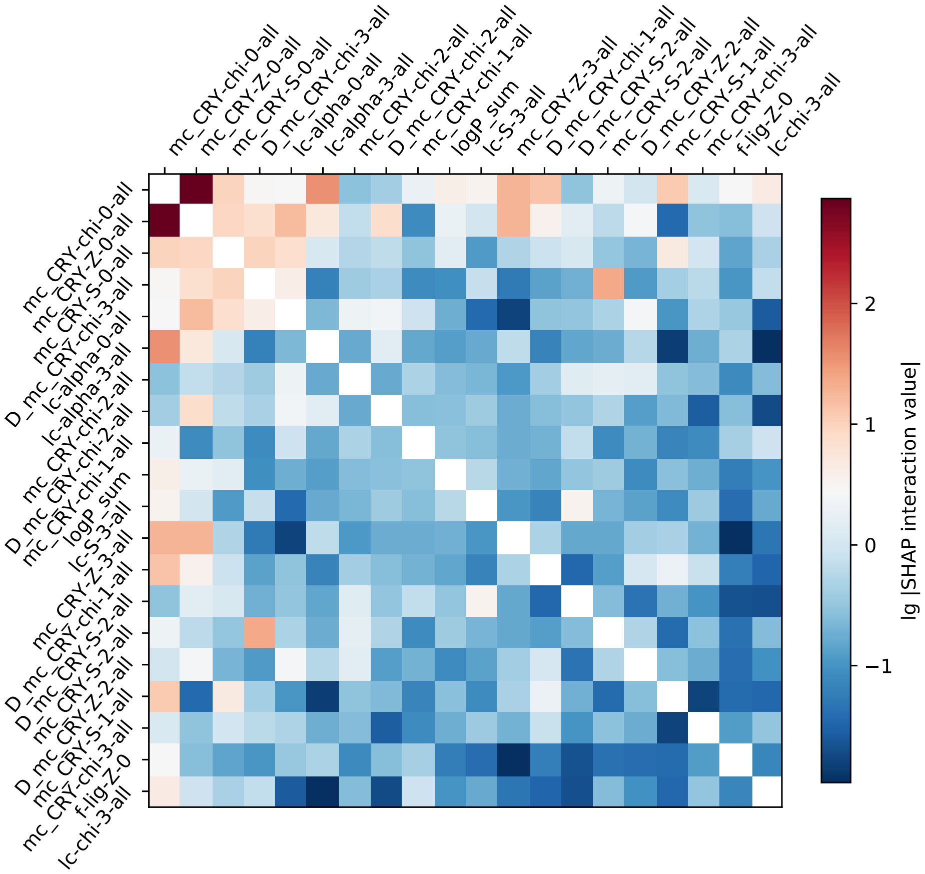

Supplementary Figure 24 | Highest absolute SHAP interaction values for the blue colour channel.

8 Colour calibration

We used the Spydercheck Checker 24 colour rendition chart with the accompanying software to create a correction profile for `Adobe Lightroom`, which we used for initial comparisons. All processing of the photos was performed in `Adobe Lightroom`, `ImageJ` or `macOS Preview`.

8.1 Semi-automatic colour calibration

Using a colour rendition chart, one can calibrate the colour—either using photo editing software like `Lightroom` or `Photoshop` together with the colour rendition chart. The most important step in this process is the correction of the white balance.

We perform the neutralisation before the colour calibration by dividing and then multiplying the image by the reference colour of the grey neutral 5 (.70 D) swatch.

8.1.1 Caveats

Colour calibration is extremely sensitive to spotlight effects, i.e., when one part of the image is much brighter than another part (see also a corresponding issue on GitHub `https://github.com/danforthcenter/plantcv/issues/254`). To detect if this is a problem one can analyse the image profile, for example, using `ImageJ` (see Supplementary Figure 25).

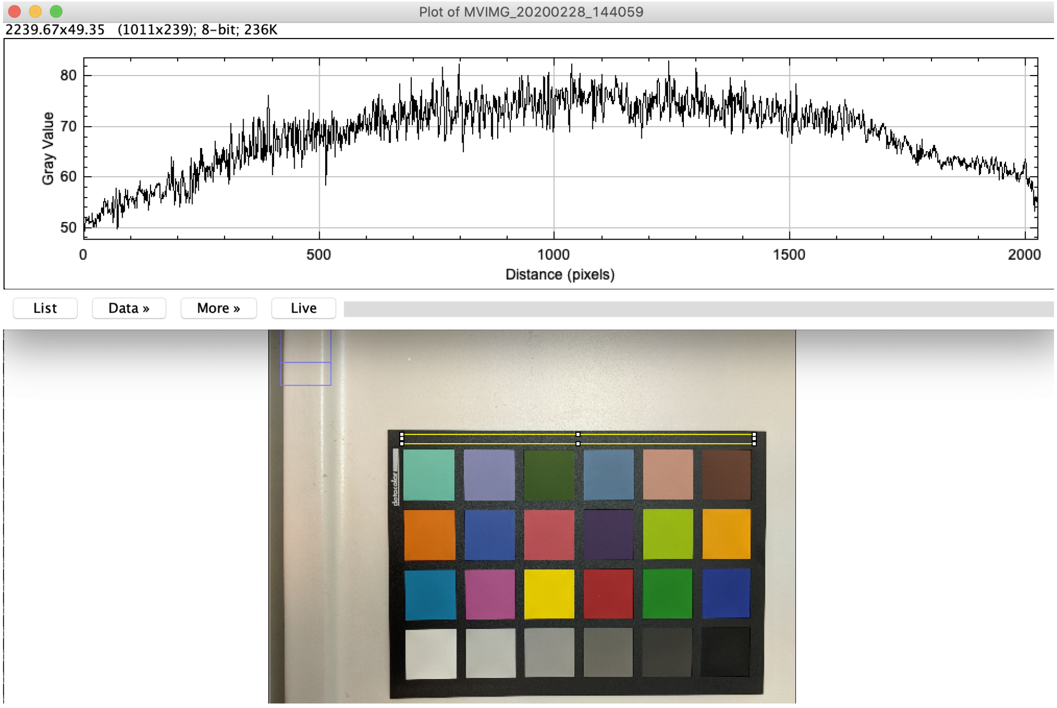

Supplementary Figure 25 | Example of non-uniform lightning that can be easily analysed with the profile function (CMD + K after selection a part of the image) in `ImageJ`. The yellow marked region is analysed for the profile.

Additionally, the Python version of the colour calibration code also outputs parity plots for which all points should fall on the diagonal (see Supplementary Figure 26). If this is not the case for only a few patches one can attempt to exclude those from the calibration. Otherwise, (see Supplementary Figure 27), we recommend retaking the photo with better lighting.

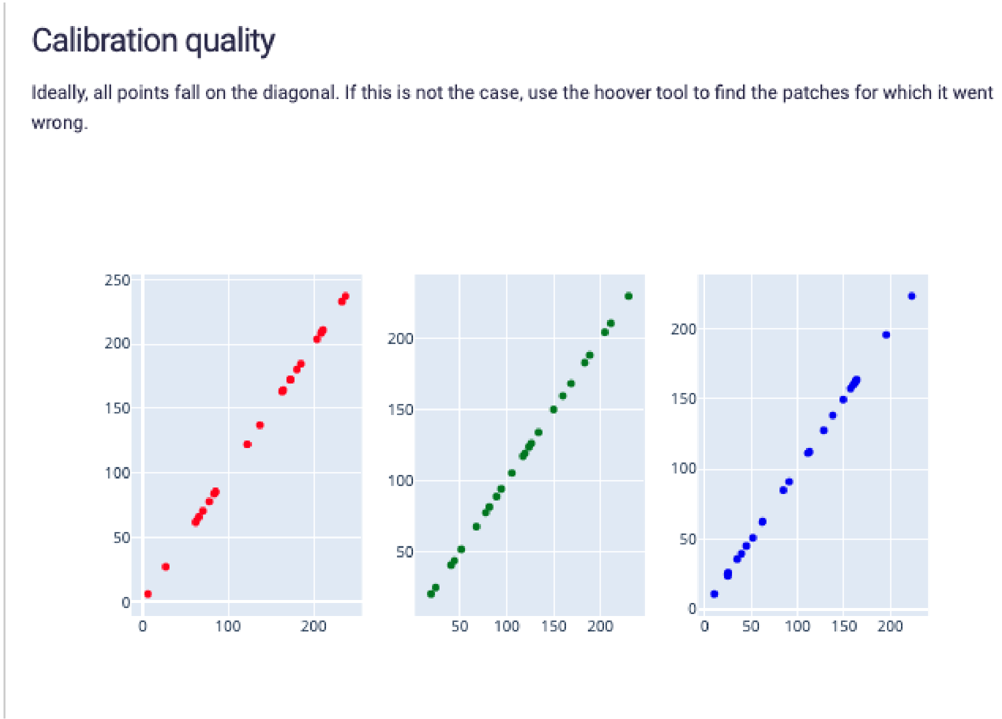

Supplementary Figure 26 | Example of the parity plot for a perfect calibration.

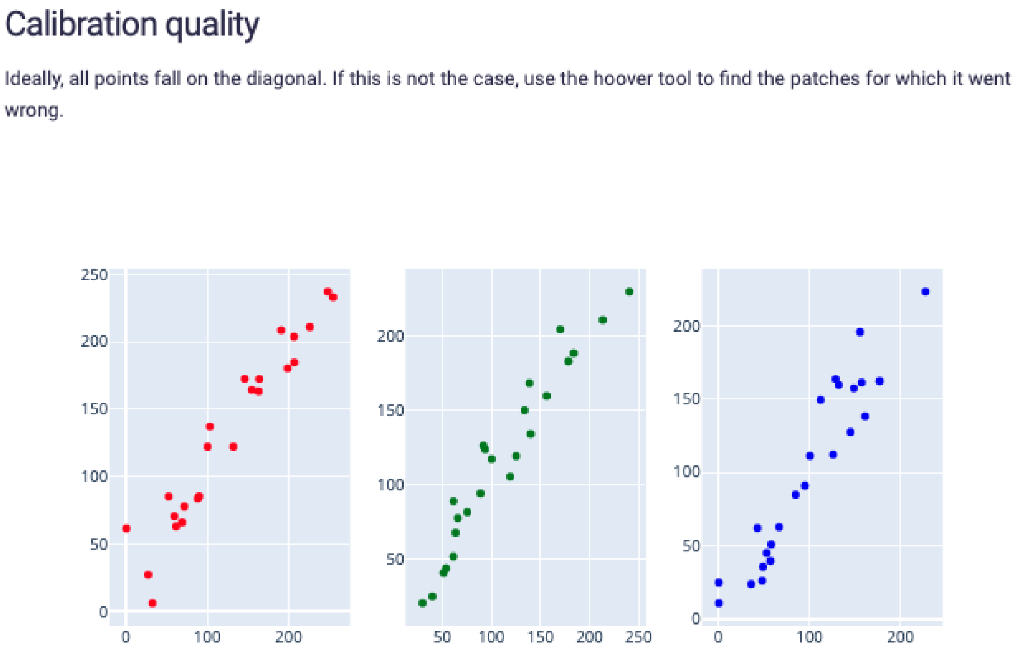

Supplementary Figure 27 | Example of the parity plot for an unreliable calibration.

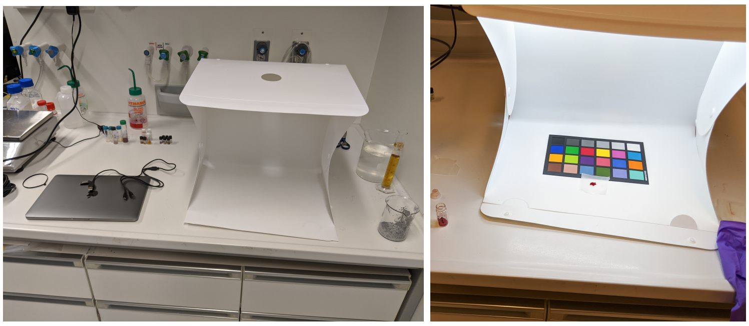

Supplementary Figure 28 | Lightbox setup used to take photographs of the powders.

In practice, spotlight effects can be avoided by means of a diffuser and by taking the photographs from some distance. We used a setup as shown in Supplementary Figure 28, where we use a lightbox to reduce shadows and ensure homogeneous illumination.

Additionally, for colour calibration, we have to keep in mind that typically the illuminant changes and is different from the reference values we use in the software (CIE D50). We are planning to investigate approaches in which the illuminant is inferred from images using machine learning (ML).[35;36]

Note also that we took some photos through the glass of a vial (e.g., when we wanted to measure the colour of an activated material). In this case, the glass can slightly distort the colour measurement. Also note that for powders the result will depend on the powder density, i.e. air to grain ratio. For this reason some recommend measuring the powder in the form of a tablet.[37] To minimise effects of light trapping[38] between the particles and increase reproducibility we found that the sample thickness should be at least half a centimetre.

Furthermore, we observed a significant variability of colour for different batches of the same material. This is also reflected in the literature, for example for Ni-MOF-74, for which both yellow-green[39] and yellow-brown[40] have been reported.

8.2 Implementation

8.2.1 Python implementation

For the Python implementation we built a Dash app and use the implementation in `colour-checker-detection` for detection of the colour rendition card.[41] We offer the user a selection of calibration algorithms (polynomial expansions using the Vandermonde method, the method proposed by Cheung et al.[42] and by Finlayson et al.[43]), which are implemented

in the `colour` Python package.[44] By default we use the algorithm proposed by Finlayson et al. Operations on the colours are performed in linear sRGB space (applying the decoding colour component transfer function (CCTF) to the non-linearly encoded images). The code is, together with a Dockerfile which we use for deployment, available on GitHub (`https://github.com/kjappelbaum/colorcalibrator`).

8.2.2 JavaScript Implementation

We are currently implementing semi-automatic colour calibration in JavaScript (ES2015). The source code is available on GitHub (`https://github.com/kjappelbaum/colorcal`). In general, our implementation follows the algorithms described by Sunoj et al.[45] For the patch selection, the user selects the edges of a colour calibration card. Using geometric reasoning, we determine the coordinates of the 24 patches of the colour calibration card—also in case of moderate tilt or bad alignment. We only assume that the full card is visible in the image. For each patch, we select a rectangular region of interest (ROI) in which we compute the average RGB colour.

9 Telegram chatbot

To facilitate the upload of photos of the synthesised compounds into the ELN,[46] we developed a prototype of a Telegram chatbot using the `PyTelegramBotAPI` Python package.[47] The code of the prototype is available on GitHub (`https://github.com/kjappelbaum/elnbot`). An example from the first interaction (asks for the EPFL username) to the upload of an image is shown in Supplementary Figure 29.

The ELN employed by us already supports automatic upload from several instruments via samba shares. For the chatbot we simply save an image with the correct filename (including the username, sample name, and batch number) on a samba share from which a script takes over to attach the image to the correct entry in the ELN via the `CouchDB`.

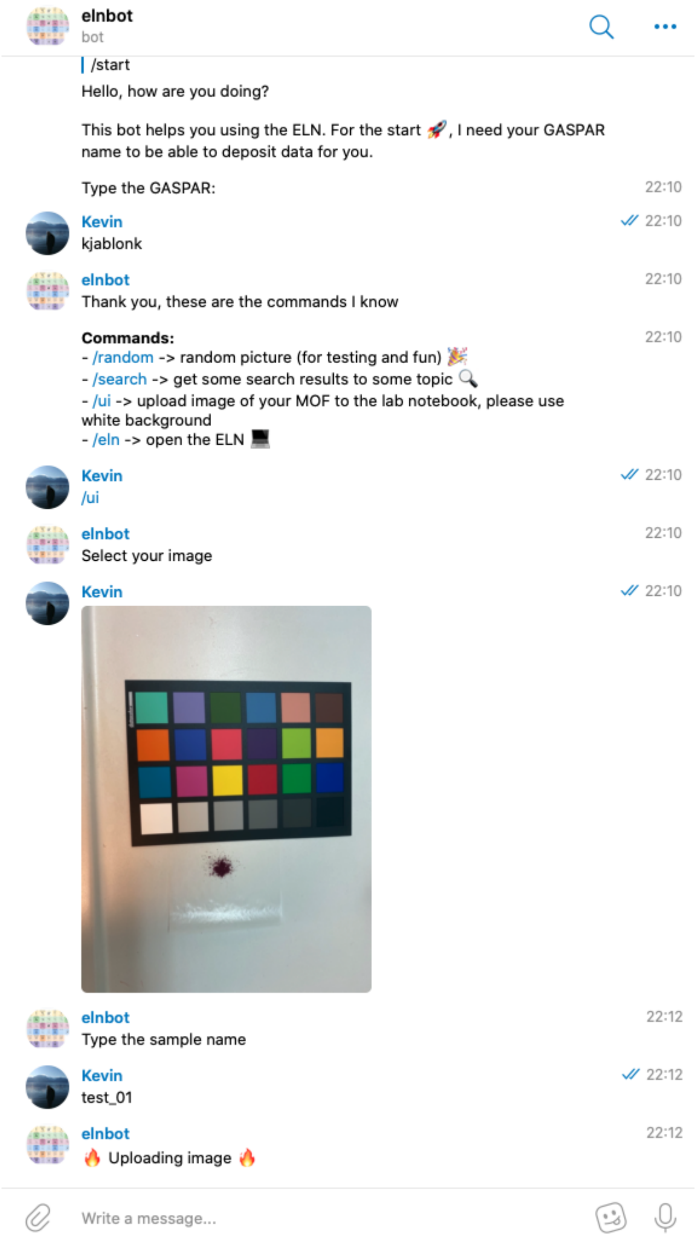

Supplementary Figure 29 | Screenshot of the interaction with the Telegram chatbot that can be used to upload an image to the ELN.

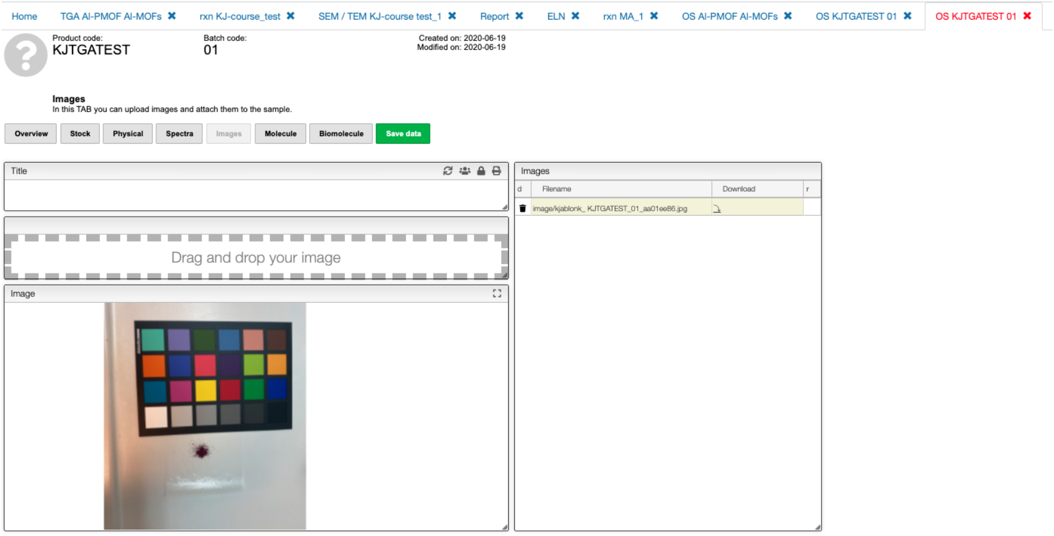

Supplementary Figure 30 | Screenshot of the image attached to a sample in the ELN.

We investigate extending the chatbot to allow interaction with more features of the ELN.

10 Web app

We implemented the web app (see Supplementary Figure 31) for the colour prediction using Dash[48] and use crystaltoolkit[49] to visualise the structures. On the GitHub repository (`https://github.com/kjappelbaum/mofcolorizer`) we provide a Dockerfile that allows building a Docker image that can be used to run this image on any platform. For example, we deploy the image on a `Dokku` instance (`https://go.epfl.ch/mofcolorizer`).[50]

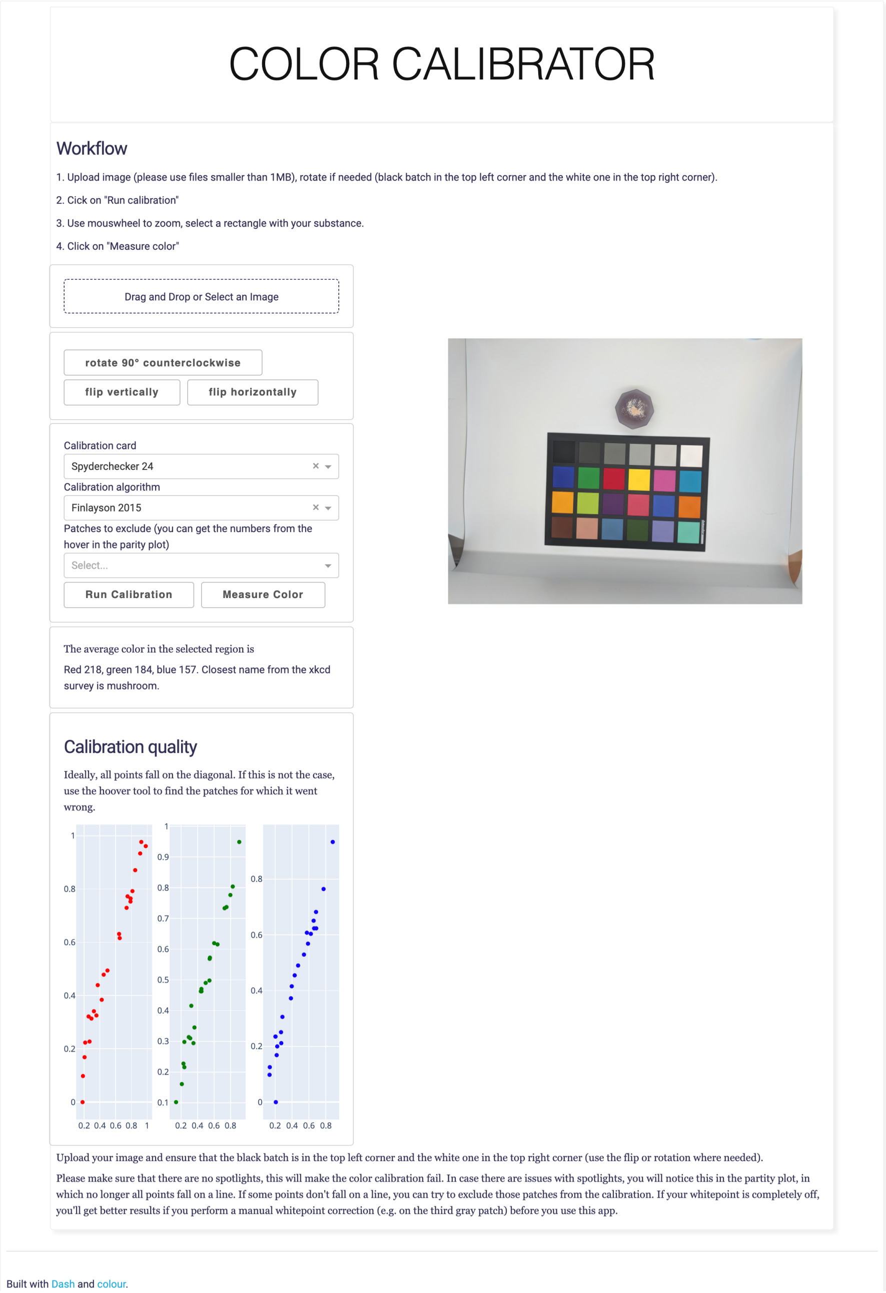

Supplementary Figure 31 | Screenshot of the web app.

References

1. Chung, Y. G.; Haldoupis, E.; Bucior, B. J.; Haranczyk, M.; Lee, S.; Zhang, H.; Vogiatzis, K. D.; Milisavljevic, M.; Ling, S.; Camp, J. S.; Slater, B.; Siepmann, J. I.; Sholl, D. S.; Snurr, R. Q. Advances, Updates, and Analytics for the Computation-Ready, Experimental Metal–Organic Framework Database: CoRE MOF 2019. *J. Chem. Eng. Data* **2019**, *64*, 5985–5998.

2. Groom, C. R.; Bruno, I. J.; Lightfoot, M. P.; Ward, S. C. The Cambridge Structural Database. *Acta Crystallogr B Struct Sci Cryst Eng Mater* **2016**, *72*, 171–179.

3. Moosavi, S. M.; Nandy, A.; Jablonka, K. M.; Ongari, D.; Janet, J. P.; Boyd, P. G.; Lee, Y.; Smit, B.; Kulik, H. J. Understanding the Diversity of the Metal-Organic Framework Ecosystem. *Nat. Commun.* **2020**, *11*, 4068.

4. Biewald, L. Experiment Tracking with Weights and Biases. 2020; `https://www.wandb.com/`, Software available from wandb.com.

5. Ke, G.; Meng, Q.; Finley, T.; Wang, T.; Chen, W.; Ma, W.; Ye, Q.; Liu, T.-Y. In *Advances in Neural Information Processing Systems 30*; Guyon, I., Luxburg, U. V., Bengio, S., Wallach, H., Fergus, R., Vishwanathan, S., Garnett, R., Eds.; Curran Associates, Inc., 2017; pp 3146–3154.

6. Natekin, A.; Knoll, A. Gradient Boosting Machines, a Tutorial. *Front Neurorobot* **2013**, *7*.

7. Bucior, B. J.; Rosen, A. S.; Haranczyk, M.; Yao, Z.; Ziebel, M. E.; Farha, O. K.; Hupp, J. T.; Siepmann, J. I.; Aspuru-Guzik, A.; Snurr, R. Q. Identification Schemes for Metal–Organic Frameworks To Enable Rapid Search and Cheminformatics Analysis. *Crystal Growth & Design* **2019**, *19*, 6682–6697.

8. Li, L.; Jamieson, K.; DeSalvo, G.; Rostamizadeh, A.; Talwalkar, A. Hyperband: A Novel Bandit-Based Approach to Hyperparameter Optimization. *arXiv:1603.06560 [cs, stat]* **2018**,

9. Wu, D.; Sun, D.-W. Colour Measurements by Computer Vision for Food Quality Control – A Review. *Trends Food Sci Technol* **2013**, *29*, 5–20.

10. Castro, W.; Oblitas, J.; De-la-Torre, M.; Cotrina, C.; Bazán, K.; Avila-George, H. *Using Machine Learning Techniques and Different Color Spaces for the Classification of Cape Gooseberry (Physalis Peruviana L.) Fruits According to Ripeness Level*; 2019.

11. Mansencal, T. et al. Colour 0.3.15. Zenodo, 2020.

12. Syzgantseva, M. A.; Stepanov, N. F.; Syzgantseva, O. A. Band Alignment as the Method for Modifying Electronic Structure of Metal-Organic Frameworks. *ACS Appl. Mater. Interfaces* **2020**, *12*, 17611–17619.

13. Cavka, J. H.; Jakobsen, S.; Olsbye, U.; Guillou, N.; Lamberti, C.; Bordiga, S.; Lillerud, K. P. A New Zirconium Inorganic Building Brick Forming Metal Organic Frameworks with Exceptional Stability. *J. Am. Chem. Soc.* **2008**, *130*, 13850–13851.

14. Millward, A. R.; Yaghi, O. M. Metal-Organic Frameworks with Exceptionally High Capacity for Storage of Carbon Dioxide at Room Temperature. *J. Am. Chem. Soc.* **2005**, *127*, 17998–17999.

15. Fateeva, A.; Chater, P. A.; Ireland, C. P.; Tahir, A. A.; Khimyak, Y. Z.; Wiper, P. V.; Darwent, J. R.; Rosseinsky, M. J. A Water-Stable Porphyrin-Based Metal-Organic Framework Active for Visible-Light Photocatalysis. *Angew. Chem. Int. Ed.* **2012**, *51*, 7440–7444.

16. Wan, Y.; Sun, W.; Liu, J.; Liu, Z. A 3D Porous Luminescent Terbium Metal-Organic Framework for Selective Sensing of F- in Aqueous Solution. *Inorg Chem Commun* **2017**, *80*, 53–57.

17. Nohra, B.; El Moll, H.; Rodriguez Albelo, L. M.; Mialane, P.; Marrot, J.; Mellot-Draznieks, C.; O'Keeffe, M.; Ngo Biboum, R.; Lemaire, J.; Keita, B.; Nadjo, L.; Dolbecq, A. Polyoxometalate-Based Metal Organic Frameworks (POMOFs): Structural Trends, Energetics, and High Electrocatalytic Efficiency for Hydrogen Evolution Reaction. *J. Am. Chem. Soc.* **2011**, *133*, 13363–13374.

18. Lei, Z.; Pei, X.-L.; Jiang, Z.-G.; Wang, Q.-M. Cluster Linker Approach: Preparation of a Luminescent Porous Framework with NbO Topology by Linking Silver Ions with Gold(I) Clusters. *Angew. Chem. Int. Ed.* **2014**, *53*, 12771–12775.

19. Cavellec, M.; Egger, C.; Linares, J.; Nogues, M.; Varret, F.; Férey, G. Oxyfluorinated Open Framework Compounds. *J. Solid State Chem.* **1997**, *134*, 349–355.

20. Zhong, Y.; Fu, H.; Meng, J.; Wang, E. Syntheses, Crystal Structures and Photochemistry of Two New Organic–Inorganic Hybrid Compounds Based on Copper–Glycin Complexes and Paradodecatungstates. *Journal of Coordination Chemistry* **2010**, *63*, 26–35.

21. Wang, X.-S.; Meng, L.; Cheng, Q.; Kim, C.; Wojtas, L.; Chrzanowski, M.; Chen, Y.-S.; Zhang, X. P.; Ma, S. Three-Dimensional Porous Metal–Metalloporphyrin Framework Consisting of Nanoscopic Polyhedral Cages. *J. Am. Chem. Soc.* **2011**, *133*, 16322–16325.

22. Herrera, J.-M.; Baca, S. G.; Adams, H.; Ward, M. D. Syntheses and Structures of Two- and Three-Dimensional Cyanide-Bridged Coordination Networks Derived from Crystallization of Diimine-Tetracyanoruthenate Anions with Gadolinium(III) Cations. *Polyhedron* **2006**, *25*, 869–875.

23. Dong, X.-Y.; Li, B.; Ma, B.-B.; Li, S.-J.; Dong, M.-M.; Zhu, Y.-Y.; Zang, S.-Q.; Song, Y.; Hou, H.-W.; Mak, T. C. W. Ferroelectric Switchable Behavior through Fast Reversible De/Adsorption of Water Spirals in a Chiral 3D Metal–Organic Framework. *J. Am. Chem. Soc.* **2013**, *135*, 10214–10217.

24. Chen, J.; Kitagawa, S. Preparation of Porous Cobalt(II) 1,3,5-Benzenetricarboxylates: Product Dependence on the Reaction Solvent and Additive. *Chem. Lett.* **2011**, *40*, 656–657.

25. Atherton, Z.; Goodgame, D. M. L.; Katahira, D. A.; Menzer, S.; White, A. J. P.; Williams, D. J. Chain and Rhomboidal "Box"-like Polymeric Metal Complexes of N′,N′-1,2-Ethylenebis(Pyrin-2thione). *Polyhedron* **1998**, *17*, 2257–2265.

26. Chainok, K.; Neville, S. M.; Moubaraki, B.; Batten, S. R.; Murray, K. S.; Forsyth, C. M.; Cashion, J. D. Synthesis, Structures and Spin Crossover Properties of Infinite 3D Frameworks of Iron(II) Containing Organodinitrile Bridging Ligands. *Dalton Trans.* **2010**, *39*, 10900–10909.

27. Johnson, J. A.; Chen, S.; Reeson, T. C.; Chen, Y.-S.; Zeng, X. C.; Zhang, J. Direct X-Ray Observation of Trapped CO2 in a Predesigned Porphyrinic Metal–Organic Framework. *Chem. Eur. J* **2014**, *20*, 7632–7637.

28. Chen, Y.-F.; Ma, Y.-C.; Zhang, H.-B.; Chen, S.-M. An Effective Approach for Constructing Acentric Heterometallic-Organic Framework with Catalytic Activity. *Inorg Chem Commun* **2014**, *43*, 27–30.

29. Wang, X.; Yan, P.; Li, Y.; An, G.; Yao, X.; Li, G. Highly Efficient White-Light Emission and UV–Visible/NIR Luminescence Sensing of Lanthanide Metal–Organic Frameworks. *Crystal Growth & Design* **2017**, *17*, 2178–2185.

30. Guo, Z.; Yan, D.; Wang, H.; Tesfagaber, D.; Li, X.; Chen, Y.; Huang, W.; Chen, B. A Three-Dimensional Microporous Metal–Metalloporphyrin Framework. *Inorg. Chem.* **2015**, *54*, 200–204.

31. Bélombé, M. M.; Nenwa, J.; Mbiangué, Y.-A.; Bebga, G.; Majoumo-Mbé, F.; Hey-Hawkins, E.; Lönnecke, P. Water-Filled Pseudo-Nanotubes in Ag11.60H0.40[Cr(C2O4)3]4·15H2O: Synthesis, Characterization and X-Ray Structure. *Inorg. Chim. Acta* **2009**, *362*, 1–4.

32. Zhang, M.-D.; Qin, L.; Yang, H.-T.; Li, Y.-Z.; Guo, Z.-J.; Zheng, H.-G. Series of Metal–Organic Frameworks Including Novel Architectural Features Based on a Star-like Tri(4-Pyridylphenyl)Amine Ligand. *Cryst. Growth Des.* **2013**, *13*, 1961–1969.

33. Ojala, M.; Garriga, G. C. Permutation Tests for Studying Classifier Performance. 2009 Ninth IEEE International Conference on Data Mining. Miami Beach, FL, USA, 2009; pp 908–913.

34. Huang, B.; Symonds, N. O.; von Lilienfeld, O. A. In *Handbook of Materials Modeling : Methods: Theory and Modeling*; Andreoni, W., Yip, S., Eds.; Springer International Publishing: Cham, 2018; pp 1–27.

35. Bianco, S.; Cusano, C.; Schettini, R. Color Constancy Using CNNs. *arXiv:1504.04548 [cs]* **2015**,

36. Hemrit, G.; Finlayson, G. D.; Gijsenij, A.; Gehler, P.; Bianco, S.; Funt, B.; Drew, M.; Shi, L. Rehabilitating the ColorChecker Dataset for Illuminant Estimation. *arXiv:1805.12262 [cs]* **2018**,

37. GmbH, C. Easy Colour Measurement of Powder. `https://www.colorlite.de/info-centre/working-with-the-spectrophotometer/how-do-i-measure-the-colour-of-powder/?lang=en/`, accessed 07.12.2020.

38. Inc, H. A. L. Measuring Loose Powder. 2012; `https://www.hunterlab.com/media/documents/mm-5039-measuring-loose-powder-with-ultrascan-vis.pdf`, accessed 07.12.2020.

39. Bonino, F.; Chavan, S.; Vitillo, J. G.; Groppo, E.; Agostini, G.; Lamberti, C.; Dietzel, P. D. C.; Prestipino, C.; Bordiga, S. Local Structure of CPO-27-Ni Metallorganic Framework upon Dehydration and Coordination of NO. *Chem. Mater* **2008**, *20*, 4957–4968.

40. Caskey, S. R.; Wong-Foy, A. G.; Matzger, A. J. Dramatic Tuning of Carbon Dioxide Uptake via Metal Substitution in a Coordination Polymer with Cylindrical Pores. *J. Am. Chem. Soc.* **2008**, *130*, 10870–10871.

41. Developers, C. Colour-Checker-Detection. colour-science, 2020.

42. Cheung, V.; Westland, S.; Connah, D.; Ripamonti, C. A Comparative Study of the Characterisation of Colour Cameras by Means of Neural Networks and Polynomial Transforms. *Coloration Technology* **2004**, *120*, 19–25.

43. Finlayson, G. D.; Mackiewicz, M.; Hurlbert, A. Color Correction Using Root-Polynomial Regression. *IEEE Trans Image Process* **2015**, *24*, 1460–1470.

44. Munroe, R. Color Survey Results. 2010; `https://blog.xkcd.com/2010/05/03/color-survey-results/`.

45. Sunoj, S.; Igathinathane, C.; Saliendra, N.; Hendrickson, J.; Archer, D. Color Calibration of Digital Images for Agriculture and Other Applications. *ISPRS Journal of Photogrammetry and Remote Sensing* **2018**, *146*, 221–234.

46. Patiny, L.; Zasso, M.; Kostro, D.; Bernal, A.; Castillo, A. M.; Bolaños, A.; Asencio, M. A.; Pellet, N.; Todd, M.; Schloerer, N.; Kuhn, S.; Holmes, E.; Javor, S.; Wist, J. The C6H6 NMR Repository: An Integral Solution to Control the Flow of Your Data from the Magnet to the Public. *Magn. Reson. Chem.* **2018**, *56*, 520–528.

47. eternnoir, pyTelegramBotAPI. 2020; `https://github.com/eternnoir/pyTelegramBotAPI`.

48. Parmer, C. Dash. Plotly, 2020.

49. Horton, M. Materialsproject/Crystaltoolkit. Materials Project, 2020.

50. Lindsay, J. Dokku/Dokku. Dokku, 2020.

Acronyms

$k$nn k-nearest neighbor. 30

BNN Bayesian neural network. 15

CCTF colour component transfer function. 43

CIELAB CIE 1976 L*a*b*. 18

CoRE Computation-Ready, Experimental. 14

CSD Cambridge Structure Database. 13, 14, 28, 29

GBDT gradient boosted decision tree. 15, 30, 32, 34

GPR Gaussian process regression. 15

HSL Hue, Saturation and Luminance. 17, 18

MAE mean-absolute error. 30, 34

ML machine learning. 42

MOF metal-organic framework. 13, 14, 19, 20

MSE mean-squared error. 30

RAC revised autocorrelation. 14

RGB red, green, blue. 4, 5, 17–20, 28, 43

ROI region of interest. 43
